# DNA as a perfect quantum computer based on the quantum physics principles

**DOI:** 10.1038/s41598-024-62539-5

**Published:** 2024-05-21

**Authors:** R. Riera Aroche, Y. M. Ortiz García, M. A. Martínez Arellano, A. Riera Leal

**Affiliations:** 1 Department of Research in Physics, University of Sonora, Hermosillo, Sonora Mexico; 2 Research Institute of Dentistry, University of Guadalajara, Guadalajara Jalisco, Mexico; 3 General Hospital of the State of Sonora, Boulevar José María Escrivá de Balaguer 157, Colonia Villa del Palmar, C.P. 83105 Hermosillo, Sonora Mexico; 4 Research and Higher Education Center of UNEPROP, Hermosillo, Sonora Mexico

**Keywords:** DNA, Oscillatory resonant quantum states, Electron and hole pairs, Josephson Junction qubit, Biophysics, Chemical biology

## Abstract

DNA is a complex multi-resolution molecule whose theoretical study is a challenge. Its intrinsic multiscale nature requires chemistry and quantum physics to understand the structure and quantum informatics to explain its operation as a perfect quantum computer. Here, we present theoretical results of DNA that allow a better description of its structure and the operation process in the transmission, coding, and decoding of genetic information. Aromaticity is explained by the oscillatory resonant quantum state of correlated electron and hole pairs due to the quantized molecular vibrational energy acting as an attractive force. The correlated pairs form a supercurrent in the nitrogenous bases in a single band $\pi$-molecular orbital ($\pi$-MO). The MO wave function $(\Phi )$ is assumed to be the linear combination of the n constituent atomic orbitals. The central Hydrogen bond between Adenine (A) and Thymine (T) or Guanine (G) and Cytosine (C) functions like an ideal Josephson Junction. The approach of a Josephson Effect between two superconductors is correctly described, as well as the condensation of the nitrogenous bases to obtain the two entangled quantum states that form the qubit. Combining the quantum state of the composite system with the classical information, RNA polymerase teleports one of the four Bell states. DNA is a perfect quantum computer.

## Introduction

The natural processes are based on codes establishing the laws and principles governing physics, chemistry, biology relationships and interactions, and the concepts of matter, space, and time. For example, our decimal number system has numbers from zero to ten. With these ten numbers, the set of all Real numbers is constituted. Similarly, the instructions in a gene that tells a cell how to make a specific protein are enclosed in our genetic code^1,2^. The protein-coding gene alphabet is based on four nitrogen heteroaromatic compounds, Adenine (A), Guanine (G), Thymine (T), and Cytosine (C), classified into two types: the purines (A and G) and the pyrimidines (C and T). The two complementary Deoxyribonucleic Acids (DNA) strands constituted by nucleotides linked by a 3'–5' phosphodiester bond are held together by the Hydrogen (H) bonds (H-bond) that arise between a purine and a pyrimidine nucleic base (A-T or G-C). This structure was proposed in 1953 by Watson and Crick^3^. However, Watson–Crick or canonical base pairs are only two of the ten possible combinations between the nitrogenous bases^4^.

Advances in DNA sequencing open prospects for rapid and reliable genome analysis, promising the establishment of personalized medicine in cancer and other health problems^5^. It is fascinating to obtain polynomial and exponential computational speedups for an efficient solution to the issues associated with DNA sequencing techniques. Quantum computing is based on a set of operations to be performed simultaneously, better known as parallelism, that does not use classical bits as the basis of computing. It uses a quantum system of two states that conform to a quantum bit (qubit)^6^. A qubit has the values zero and one and the overlap of the binary states. The most general normalized state can be expressed as a linear combination of these values: $A|0\rangle +B|1\rangle$, where A and B are complex numbers that satisfy ${\left|A\right|}^{2}+{\left|B\right|}^{2}=1$, and the overall phase is physically irrelevant^7^.

The smallest nontrivial complex Hilbert space is two-dimensional $\text{U}=\left[|0\rangle ,|1\rangle \right]$, and contains qubits as its unit vectors^8^. The qubit measurement mechanism is done concerning the orthonormal basis. In this case, the outcome $|0\rangle$ is ${\left|A\right|}^{2}$, and the outcome $|1\rangle$ is ${\left|B\right|}^{2}$^9^. A qubit vector state unit is denoted as $$|0\rangle =\left[\begin{array}{c}1\\ 0\end{array}\right]$$ and $$|1\rangle =\left[\begin{array}{c}0\\ 1\end{array}\right]$$. The direct product of the Hilbert spaces of each constituent subsystem gives the structure of the Hilbert space in a composite system. In contrast, the measurements associated with each subsystem will act exclusively on its corresponding Hilbert space^10^. The qubits can operate simultaneously on all possible binary input strings of any length n, where n is the system number of a qubits in the form of ${2}^{\text{n}}$. Thus, the Hilbert space grows exponentially with the number of particles^11^.

Generally, any quantum system with two well-defined states is enough to create a qubit^11^. For example, if we talk about the spin of an electron, its states could be linear combinations of the spin up $\left(\uparrow \right)$ or down $\left(\downarrow \right)$: $\alpha \left|\uparrow \rangle \right.+\beta \left|\downarrow \rangle \right.$. Some quantum systems well characterized in which their electronic states represent the qubit states are ion or atom traps^12^, quantum dots^13^, the nuclear spins of one or several molecules^6^, and the superconducting loops with a persistent current^6^. DNA cryptography is a newborn cryptographic field that emerged with the research of DNA computing, in which the biological polymer is used as an information resource and modern biological technology as an implementation tool^14^. Our understanding of the physics of biological molecules, such as DNA, is limited because of the configurational complexity of biomolecules. We cannot establish efficient algorithms even with the best current supercomputing facilities.

Manipulations of the quantum systems include gate operations^6^, information storage^15^, protection against the effects of noise^16^, the creation of entanglement to teleportation^17^, and entanglement swapping^18^. Although a qubit can vary continuously between a set of quantum states, it can assume a single deterministic state as a single classical bit after its measurement. The process can only be applied once since it loses its superposition when a qubit is measured^19^. The power of quantum computing is brought about by its inherent parallelism and entanglement. It is possible, for example, given a function f, to simultaneously evaluate f(x) for many x values with the simple application of a quantum gate^20^. Any quantum circuit can be simulated with arbitrary precision using a combination of Controlled gates (C-Gates), such as the controlled-NOT (CNot) gate and qubit rotations^21^.

Although considerable progress has been made in understanding quantum dynamic processes, a fully comprehensive analogy in DNA analysis has yet to be found. One big difference between molecular materials and extended inorganic solids lies in the ability to have relatively weak interactions between the molecular networks, primarily due to intermolecular contacts such as Van der Waals forces or H- bonds^22^. Here, we apply physics approximations to demonstrate theoretically that A-T and C-G are maximally entangled quantum states and could be examples of two superconductors coupled together, like in some solids. A Josephson Junction could be formed with two molecular superconductors (A-T and C-G) connected to a central H-bond. The electric supercurrent generated by the electron pairs confined in the canonical base pairs $\pi$-cloud is due to the formation of oscillatory resonant quantum states between electron and hole pairs. In our model approach for genetic informatics, we represent qubits based on different pairs of binary-oppositional indicators of A, G, C, and T. Using quantum parallelism to mimic the way current classical parallel algorithms work, here we speculate about how information is obtained by reading a given gene. To understand biology at the molecular level, it is necessary to relate the complex structure, the diverse chemistry, and the traditional concepts from quantum-solid-state physics principles. God created the perfect quantum computer: the DNA.

## DNA alphabet structure

DNAs are polymers made of discrete building blocks that impart functional specificity. Four nitrogen (N) heteroaromatic compounds are combined in our DNA to form triplets to code for an amino acid^2,23^. The two fused rings that compose purines have four N^24^. In one of them, the lone pair is delocalized and is part of the $\pi$ electron system of the aromatic ring^25^. Pyrimidines are organic compounds like Benzene and pyridine but with two N atoms that replace Carbon in positions one and three^24^. Delocalized $\pi$ electron clouds of aromatic residues are known to be involved in $\pi$-$\pi$ interactions^26^.

Insights into the nature of non-covalent interactions and nucleic acid bases' stabilization energy/ enthalpy explain the resulting structure and stability. The A-T and C-G pairings form double and triple H-bonds between the amine and carbonyl groups. The aromatic stacking involving the delocalized $\pi$ electrons of the rings is also crucial^4^. The $\pi$-$\pi$ interaction is conceptually similar to the stacking of two Benzene molecules and $\pi$–$\pi$ alignment where most of the ring-plane area overlaps is found in only a limited number of structures^27^. The pairing of purines and pyrimidines may result partly from dimensional constraints, as the combination allows for a constant-width geometry for the DNA spiral helix. Watson–Crick base pairs allow the DNA helix to maintain a regular helical structure with an approximately 2.0 nm^3^ diameter. Other combinations seem less possible; for example, stacking between two protonated C is repulsive^28^. The repulsion is evident in DNA triplexes, where two consecutive protonated C are not tolerated, sharply destabilizing the formation of Pyr-Pur-Pyr triplexes with two or more successive G in the second strand stabilization enthalpies^29,30^.

The complexity of the interplay of accurate intrinsic interaction energies in nucleic acids is so variable that one interaction may have a strikingly different effect on stability in stacking across all the specific structures. Interstrand and intrastrand stacking in a nucleic acid’s double helices are salient examples of how non-covalent interactions are of primary importance in biology^4^. Also, an intramolecular base pair can occur within single-stranded nucleic acids. In Ribonucleic Acid (RNA), the base pairs A-U and C-G allow the formation of short double-stranded helices. Non-canonical base pairing and other H-bond interactions, such as A-A, U-U, C-C, G-G, A-C, A-G, U-C, and U-G, have been described in RNA, contributing to the adoption of specific three-dimensional (3D) structures^31^. The nucleobase Uracil (U) usually takes the place of T in RNA and differs from it by lacking a methyl group on its ring^32^.

The mechanism of single- or multiple-proton transfer in DNA bases has been investigated to explain how the pair of bases are formed^33^. Nevertheless, the analysis of available published data fails to give decisive evidence. In opposition to Watson and Crick’s paired bases, A-A, C-C, G-G, T-T, A-C, A-G, T-C, and T-G have chemical structures that differ in the position of the H atom^34^. Gorb and coworkers suggested that non-Watson and Crick’s paired bases are the products of intra- and/ or intermolecular proton transfer^35^. Canonical paired bases self-assembly could constitute a case of a hetero-Diels–Alder reaction in which the Woodward-Hoffmann rules would apply to $\pi$ systems involving heteroatoms, such as carbonyls and imines, which provide the corresponding heterocycles. Organic reactions that obey these rules are allowed by symmetry^23,36^.

Biological DNA is mainly in B form, with the neighboring base pairs having an average separation of about 3.4 Å and a relative twist angle of around 36° about the helical axis^37^. This structure is regular, with the base pairs having a substantial geometrical overlap (perfectly stacked)^38^. Another double helix may be found defining the grooves between the double strands. These spaces are adjacent to the base pairs and may provide the transcription factors' specific sequences of binding sites^39^. As the strands are not symmetrically located around each other, they are unequally sized. The major groove is 2.2 nm wide, while the minor groove is 1.2 nm wide^3,40^. Some conditions confer DNA double strands stability: the G-C content and the length of the specific sequence^41^. Due to the high A-T content, the TATAAT Pribnow box in some promoters makes the strands easier to split^42^.

## Lone pairs and groups of electrons in the nitrogenous nucleobases as Cooper pairs: oscillatory resonant quantum state between *electron* and hole pairs

In a conducting material, the electrical properties are due to the most energetic electrons close to the Fermi energy ($${E}_{F})$$. In a Cooper pair, an attractive interaction between two electrons at the Fermi level produces a bound state with a total energy of less than $$2{E}_{F}$$^43^. The electron affinity is explained by Cooper^44^, considering the screen of the electron–electron interaction by a total dielectric constant due to phonons and electrons. It is imposed as a condition that the phonon energy needs to be greater than the energy difference between the electron pairs. Thus, the shielded Coulomb repulsion becomes negative, interpreting this sign as an attraction between the two electrons. These pairs are the carriers of superconductivity in the BCS (J. Bardeen, L.N. Cooper, and J.R. Schrieffer) Theory and are responsible for the gap in the energy spectrum^45^.

Aromaticity is related to cyclic $\pi$ electron delocalization in closed circuits, giving rise to energy stabilization, bond length equalization, large magnetic anisotropies, and unique chemical properties^46^. In cyclic systems, the concept of permutations, which permute electrons circularly around the ring, has been described^47^. Ring permutations were named, often becoming the most significant terms following the nearest-neighbor transposition^48^.

The notion of "hole" was developed by Werner Heisenberg in 1931 as the absence of an electron in the valence band^49^. It is a helpful way to analyze the movement of many electrons, considering a hole as a quasiparticle^50^. The electron–hole has absolute values of the same charge, but unlike the electron, it is positive^51^. The unoccupied orbitals act like a hole.

In the classical Valence Bond (VB) theory, aromaticity is explained by the resonance between Kekule structures^47^. Considering the presence of conjugated circuits in the nitrogenous base pair, we explain aromaticity by the oscillatory resonant quantum states between electron and hole pairs. In this work, like Cooper Pairs, electron and hole pairs are formed in the nitrogenous bases, not by the electron–phonon-electron relationship established by Cooper, but by the electron-vibrational energy-electron interaction. Electrons in molecules experience a direct Coulomb interaction between themselves and Coulombic interaction with the atomic nuclei. The last takes the form of the vibronic interaction^52^. In this work, we considered the Cooper phonon as a Biology Boson ${(B}_{b}$), represented by the quantized molecular vibrational energy.

**Figure 1:**
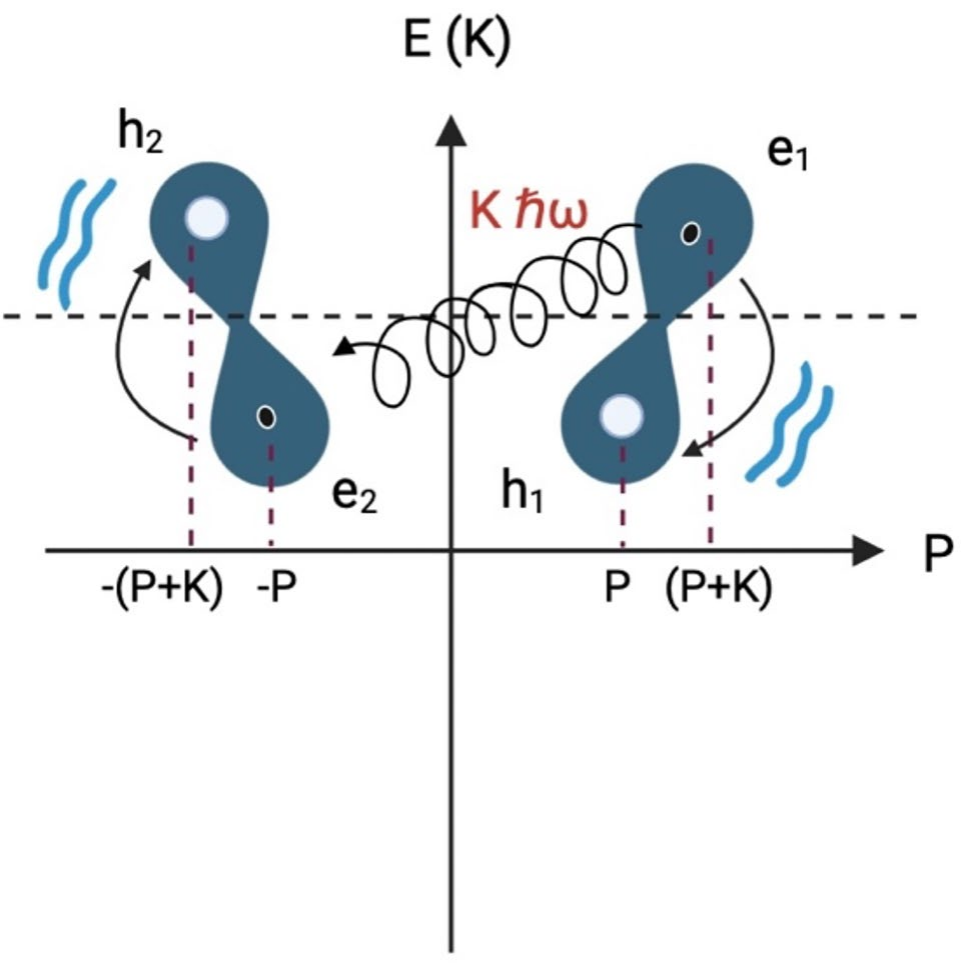
The oscillatory resonant quantum state between electron and hole pairs. The general model of the electron pairs formation in the presence of hole pairs. The attractive force between electrons and hole pairs is the quantized molecular vibration (${B}_{b}=\hslash \omega )$. Electrons exchange ${B}_{b}$. The energy difference between electrons and holes is ${B}_{b}$. The total momentum is $\pm K.$

In Fig. 1, we described the general oscillatory resonant quantum states process between electron and hole pairs forming in $\pi$ orbitals. The overlap of two ${p}_{z}$ orbitals brings two electrons with equal spin closer. We now have two interactions: the Coulombian repulsion and the electron-vibrational energy-electron. Due to the Coulombian repulsion, electron one (${e}_{1}$) is forced to occupy the position of hole one (${h}_{1}$) with momentum $P$ and energy $E\left(P\right)$ and emits ${B}_{b}$ to electron two (${e}_{2}$). The ${e}_{2}$ absorbs ${B}_{b}$ and moves to the hole two (${h}_{2}$) position with momentum $-P-K$ and energy $E\left(-P-K\right)$. In the second half of the oscillation, ${e}_{1}$ and ${e}_{2}$ exchange ${B}_{b}$ to return to their original positions (Fig. 1). The energy difference between the electrons is the same as ${B}_{b}$. That is the condition for the electron and hole pairs, one below and one above the Fermi level with opposite momentum, to oscillate. The electrons cannot occupy other states, so they do not interact with other atoms in the molecule. The resistance or dispersion energy is canceled. The wave function for the correlated pair (${e}_{1},{e}_{2}$) in the oscillatory resonant quantum state between electron and hole pairs in the momentum representation is $|P+K;-P\rangle$, and for the holes pair (${h}_{1}$,${h}_{2}$) is $|-P-K; P\rangle$.

Where: K is the momentum of ${B}_{b}$ and $\hslash \omega$ its energy.

The difference in the total momentum of the electron and hole pairs is given by:

$$P+K+P=2P+K\text{ for the electron pairs and}$$

$$-(P+K)-P=-2P-K\text{ for the hole pairs}$$

The sum of the total momentum of the electron and hole pairs is given by:

$$P+K-P=K \text{ for the electron pairs and}$$

$$-(P+K)+P=-K\text{ for the hole pairs}$$

Due to the Molecular Orbital (MO) description of Benzene providing a more satisfying and general treatment of "aromaticity"^53,54^, we will first analyze its structure using the oscillatory resonant quantum state between electron and hole pairs. The six-membered ring in Benzene is a perfect hexagon with all Carbon–Carbon bonds having an identical length of 139 pm^55^. All Carbons are ${sp}^{2}$ hybridized, and have an unhybridized ${p}_{z}$ orbital perpendicular to the ring plane^56^. When the phases correspond, the six overlap equally with both adjacent orbitals to generate a common region of a like phase, with those orbitals having the most significant overlap being the lowest in energy^53,57^. The remaining Carbon valence electrons then occupy these molecular orbitals in pairs, resulting in a fully occupied (six electrons) set of bonding molecular orbitals^53^. This closed shell gives the Benzene ring its thermodynamic and chemical stability, just as a filled valence shell octet confers stability on inert gases^55^. We call this common region $\pi$-MO of Benzene ($\pi$-MO_B_).

**Figure 2:**
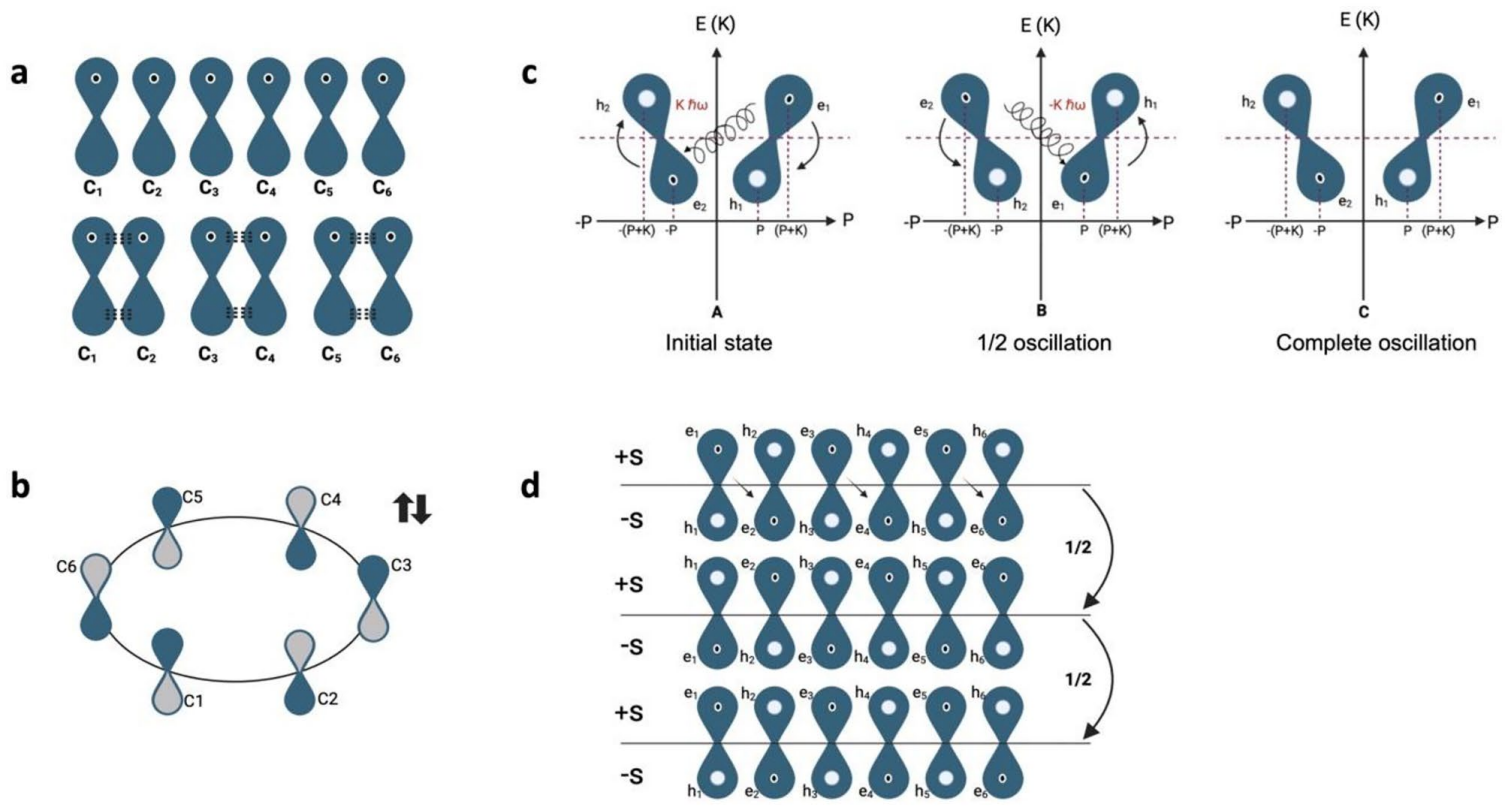
The Benzene molecule's oscillatory resonant quantum state between electron and hole pairs. (**A**) Non-hybridized in-face ${p}_{z}$ orbitals of the doubly bonded Carbon–Carbon overlap. (**B**) Readjustment of the molecular geometry, generating the same distance of Carbon–Carbon bonds. (**C**) Oscillatory resonant quantum states between electron and hole pairs to explain aromaticity in the Benzene molecule. (**D**) The diagram shows the arrangement of an annular current in the electronic structure of Benzene's ground state wave function.

When $\pi$-MO_B_ is formed and according to the Valence Shell Electron Pair Repulsion (VSEPR) Theory model, the negatively charged regions will repel each other, causing them (and therefore the chemical bonds) oriented to be as spaced apart as possible to minimize repulsions^58^. Thus, in Benzene, three unions are formed because the non-hybridized ${p}_{z}$ orbitals of the doubly bonded Carbon–Carbon overlap, forcing the electrons to be distributed in pairs with opposite spins^58^. At the same time, the repulsion interaction forces a readjustment of the molecular geometry, generating the same distance of Carbon–Carbon bonds (Fig. 2a,b). Overlapping ${p}_{z}$ orbitals on adjacent atoms form an extended $\pi$-bonding system.

Pairwise correlation condition: The energy difference between electron and hole pairs is equal to the quantized lattice vibration, ${B}_{b}$ with ${B}_{b}=\hslash \omega$, and ${E}_{{e}_{1}}-{E}_{{e}_{2}}=\hslash \omega$, ${E}_{{e}_{3}}-{E}_{{e}_{4}}=\hslash \omega$, and ${E}_{{e}_{5}}-{E}_{{e}_{6}}=\hslash \omega$. Starting from the initial state A, in a half oscillation, the electron ${e}_{1}$ emits ${B}_{b}$ to ${e}_{2},$ producing its movement toward the hole h_2_. Electrons ${e}_{3}$ and ${e}_{5}$ emit a ${B}_{b}$ to electrons ${e}_{4}$ and ${e}_{6}$_,_ respectively. Thus, in half of the oscillation, we get the state B. Then, the electrons ${e}_{2}$, ${e}_{4}$, and ${e}_{6}$ emit to ${e}_{1}$, ${e}_{3}$_,_ and ${e}_{5}$_,_ recovering the initial state A (Fig. 2c,d). We call this movement that explains the correlation of the pairs: oscillatory resonant quantum state between electron and hole pairs in Benzene.

The internal state of electron and hole pairs using the Schrödinger equation and following the formula of Riera et al.^59^ and BCS in the formation of the Cooper pairs in the Superconductivity Theory^43,45^, is represented by:

${H}_{INT}:$ Hamiltonian of the internal state of the pair equivalent to the binding energy.

$|{\psi }_{xx{\prime}}\rangle$: Wave function of the correlated electrons pairs in $\pi$-MO, where $xx{\prime}$ identifies the correlated electrons. For example, $|{\psi }_{{e}_{1}{e}_{2}}\rangle$, correlated pair (${e}_{1}$,${e}_{2}$) wave function.

$|{\psi }_{yy{\prime}}\rangle$: Hole wave function, where $yy{\prime}$ identifies the correlated hole pairs.

${H}_{INT}={U}_{{e}_{1}{e}_{2}}+{U}_{{e}_{1}{B}_{b}{e}_{2}}$, where ${U}_{{e}_{1}{e}_{2}}$ represents the Coulomb repulsion interaction potential and ${U}_{{e}_{1}{B}_{b}{e}_{2}}$ represents the potential of electron-${B}_{b}$-electron interaction with ${e}_{1}$ that emits the energy of ${B}_{b}$ to ${e}_{2}$ that absorbs it.

1 $${H}_{INT}\left|{\psi }_{\left({e}_{1,}{e}_{2}\right)}\rangle =\left({U}_{{e}_{1}{e}_{2}}+{U}_{{e}_{1}{B}_{b}{e}_{2}}\right)\right|{\psi }_{({e}_{1},{e}_{2}}\rangle =\left({E}_{{e}_{1}{e}_{2}}+{E}_{{e}_{1}{B}_{b}{e}_{2}}\right)|{\psi }_{\left({h}_{1}, {h}_{2}\right)}\rangle$$

We have here the half oscillation of the pair.

${E}_{{e}_{1}{e}_{2}}$ is the Coulomb repulsive energy and it is commutative: ${E}_{{e}_{1}{e}_{2}}={E}_{{e}_{2}{e}_{1}}$.

Equation (1) corresponds to the process of interaction electron-${B}_{b}$-electron ${e}_{1}{B}_{b}{e}_{2}$ passing the system from state A to state B.

In a complete oscillation, it goes from state B to A, but it is e_2_ that emits the ${B}_{b}$ to e_1_ that absorbs it:

$${{H}^{*}}_{INT}={U}_{{e}_{2}{e}_{1}}+{U}_{{e}_{2}{B}_{b}{e}_{1}}$$

$${{H}^{*}}_{INT}{H}_{INT}={\Delta }^{2}$$

The square of the binding energy

$${{H}^{*}}_{INT}{H}_{INT}|{\psi }_{\left({e}_{1,}{e}_{2}\right)}\rangle =\left({E}_{{e}_{1}{e}_{2}}+{E}_{{e}_{1}{B}_{b}{e}_{2}}\right)\left({U}_{{e}_{2}{e}_{1}}+{U}_{{e}_{2}{B}_{b}{e}_{1}}\right)|{\psi }_{({h}_{1}, {h}_{2})}\rangle =\left({E}_{{e}_{1}{e}_{2}}+{E}_{{e}_{1}{B}_{b}{e}_{2}}\right)\left({E}_{{e}_{2}{e}_{1}}+{E}_{{e}_{2}{B}_{b}{e}_{1}}\right)|{\psi }_{\left({e}_{1}{e}_{2}\right)}\rangle$$

We have here the complete oscillation of the pairs

${E}_{{e}_{1}{e}_{2}}={E}_{{e}_{2}{e}_{1}}$, and ${U}_{{e}_{1}{B}_{b}{e}_{2}}=-{U}_{{e}_{2}{B}_{b}{e}_{1}}$ because in the first transition, e_1_ emits and in the second, it absorbs. Then,

2 $${\Delta }^{2}= \left({E}_{{e}_{1}{e}_{2}}+{E}_{{e}_{1}{B}_{b}{e}_{2}}\right)\left({E}_{{e}_{1}{e}_{2}}-{E}_{{e}_{1}{B}_{b}{e}_{2}}\right)\Rightarrow {\Delta }^{2}={{E}^{2}}_{{e}_{1}{e}_{2}}-{{E}^{2}}_{{e}_{1}{B}_{b}{e}_{2}}$$

Equation (2) is the same obtained in BCS^45^ and the work by Riera et al.^59^ on the Theory of Superconductivity: a quadratic dispersion law used by Riera to get the Buckingham equation in BCS. Riera’s work introduced the qualitative parameter^59^.

**Figure 3:**
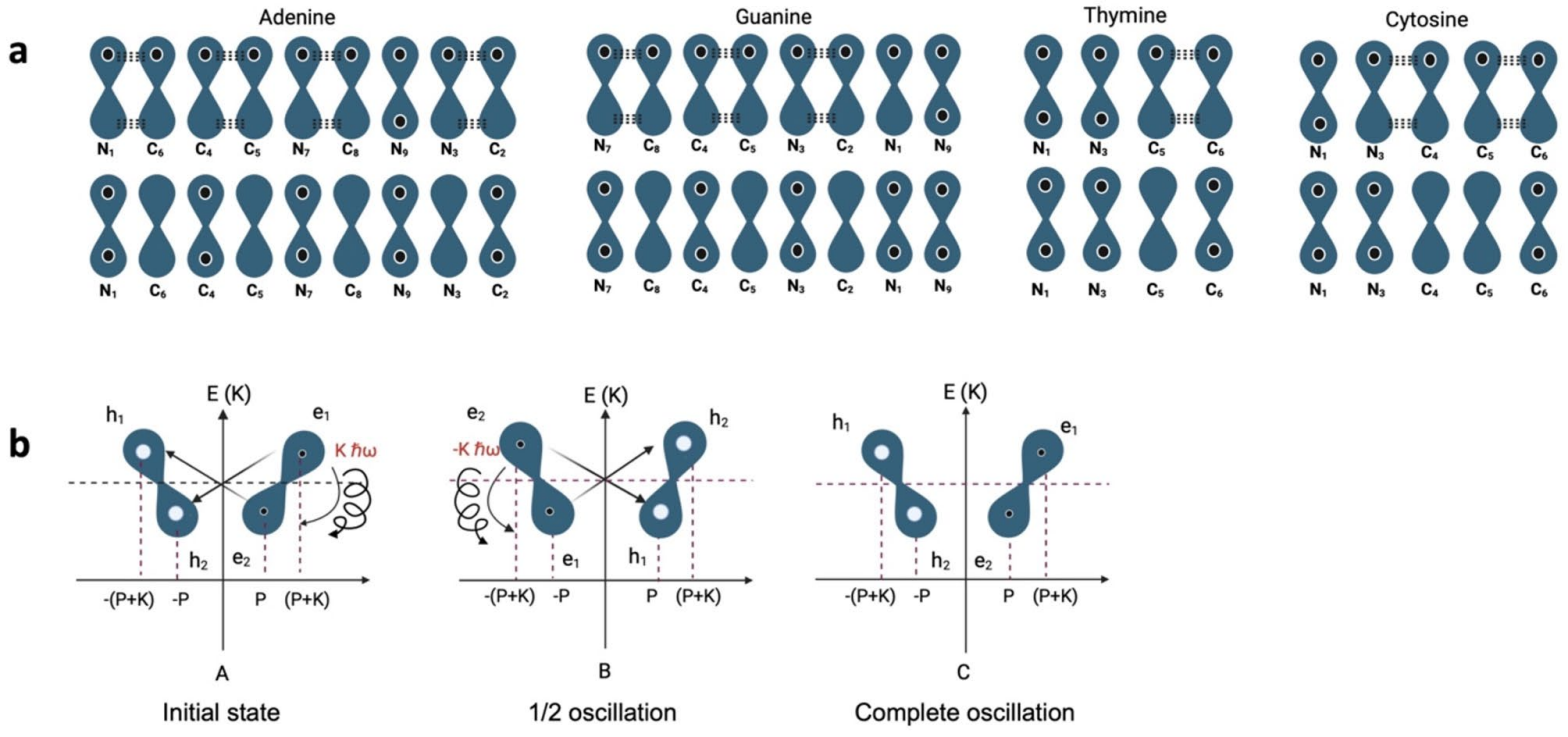
The Nucleobases A, T, C, and G oscillatory resonant quantum state between electron and hole pairs. (**A**) The non-hybridized in-face ${p}_{z}$ orbitals overlap. A lone pair of delocalized electrons will participate in resonance with the double bond and form an extended $\pi$-system. (**B**) Oscillatory resonant quantum states between electron and hole pairs to explain aromaticity in the nitrogenous bases.

Like Benzene, the nucleic acid bases are all aromatic systems. However, the orbital arrangement of electrons differs from that of Benzene in the presence of N atoms with ${p}_{z}$ orbitals containing a lone pair. A lone pair of delocalized electrons will participate in resonance with the double bond and form an extended $\pi$-system^60,61^ (Fig. 3a).

Analyzing the pairwise correlation for the nitrogenous bases:

Pairwise correlation condition: 1-There must be both electron and hole pairs. 2-The difference in energy between the correlated electron and hole pairs is equal to the quantized vibrational energy, ${B}_{b}=\hslash \omega$.

${H}_{INT}:$ Hamiltonian of the internal state of the pair equivalent to the binding energy.

$\Delta$: Pair binding energy.

${U}_{{e}_{x}{e}_{x}}$: Coulomb repulsive interaction energy.

$${U}_{{e}_{x}{B}_{b}{e}_{-x}}$$: Interaction energy in the $$x$$ orbital between the electrons $$({e}_{x})$$ with spin $$\frac{1}{2}$$ that emits $${B}_{b}$$ to the electron ($${e}_{-x})$$ with spin $$-\frac{1}{2}$$. Then, the $${e}_{x}$$ electron loses energy and moves to occupy the hole $${h}_{y}$$ and becomes $${e}_{y}$$ leaving the hole $${h}_{x}$$ (Fig. 3b).

$$\text{Then},{H}_{INT}={U}_{{e}_{x}{e}_{x}}+{U}_{{e}_{x}{B}_{b}{e}_{-x}}\text{and }{{H}^{*}}_{INT}={U}_{{e}_{y}{e}_{-y}}+{U}_{{e}_{y}{B}_{b}{e}_{-y}}$$

${\psi }_{x\left(-x\right)}=|{\psi }_{x\left(-x\right)}\rangle$: electron pair wave function in $x$ orbital.

${\psi }_{y\left(-y\right)}=|{\psi }_{y\left(-y\right)}\rangle$: hole pair wave function in $y$ orbital

3 $${H}_{INT}|{\psi }_{x\left(-x\right)}\rangle =\left({U}_{{e}_{x}{e}_{x}}+{U}_{{e}_{x}{B}_{b}{e}_{-x}}\right)|{\psi }_{x\left(-x\right)}\rangle =\left({E}_{{e}_{x}{e}_{-x}}+{E}_{{e}_{x}{B}_{b}{e}_{-x}}\right)|{\psi }_{y\left(-y\right)}\rangle$$

This would be a half oscillation of the pair. To recover the initial wave function $|{\psi }_{x\left(-x\right)}\rangle$ we must apply ${{H}^{*}}_{INT}$ again to the Eq. (3)

$${{H}^{*}}_{INT}{H}_{INT}|{\psi }_{x\left(-x\right)}\rangle =\left({E}_{{e}_{x}{e}_{-x}}+{E}_{{e}_{x}{B}_{b}{e}_{-x}}\right) \left({U}_{{e}_{y}{e}_{-y}}+{U}_{{e}_{y}{B}_{b}{e}_{-y}}\right)|{\psi }_{y\left(-y\right)}\rangle =\left({E}_{{e}_{x}{e}_{-x}}+{E}_{{e}_{x}{B}_{b}{e}_{-x}}\right)\left({E}_{{e}_{y}{e}_{-y}}+{E}_{{e}_{y}{B}_{b}{e}_{-y}}\right)|{\psi }_{x\left(-x\right)}\rangle ,\text{ but}$$

${{H}^{*}}_{INT}{H}_{INT}={\Delta }^{2}$ and ${E}_{{e}_{x}{e}_{-x}}={E}_{{e}_{y}{e}_{-y}}$. Because the Coulomb repulsion does not change, the charge is commutative. But, ${E}_{{e}_{x}{B}_{b}{e}_{-x}}=-{E}_{{e}_{y}{B}_{b}{e}_{-y}}$ because the electron first emits and then absorbs the same energy.

Then,

$${\Delta }^{2}= \left({E}_{{e}_{x}{e}_{-x}}+{E}_{{e}_{x}{B}_{b}{e}_{-x}}\right)\left({E}_{{e}_{y}{e}_{-y}}+{E}_{{e}_{y}{B}_{b}{e}_{-y}}\right)={\Delta }^{2}= \left({{E}^{2}}_{{e}_{x}{e}_{-x}}-{{E}^{2}}_{{e}_{x}{B}_{b}{e}_{-x}}\right)$$

This equation represents the dispersion law obtained in the BCS Theory and Riera et al.^45,59^.

## A-T and C-G bases pairs as coordination complexes: formation of a single molecular orbital

A coordination complex comprises a central atom or ion named the coordination center and a surrounding array of bound molecules known as ligands or complexing agents. The coordination bond is commonly a little weaker than a typical covalent bond. The central atom and all ligands constitute the coordination sphere. The number of molecules attached is called the coordination number^62–64^. The two-electron clouds in a simple triatomic molecule will stretch in opposite directions. The orbitals containing the various bonding and nonbonding pairs in the valence shell will extend from the central atom in approaches that minimize their mutual repulsions^65^. An angular separation of 180° produced a molecule with a linear geometry^66^.

The MO Theory (MOT) is a method of determining the chemical bond in which the electrons move under the influence of the nuclei of the whole molecule in delocalized orbitals^67^. MO wave function $(\Phi )$ is assumed to be the linear combination of the n constituent atomic orbitals (${X}_{\mathcal{i}}$), according to ${\Phi }_{j}=\sum_{i=1}^{n}c_{ij}X_{i}$^64^. The $c_{ij}$ coefficients can be determined numerically by substituting this equation into the Schrödinger one^68^. The electrons are delocalized as electrons occupy an orbital that is delocalized over the two atomic centers. In the MO wave function, the electrons have the same probability of being in all the available modes of distribution^1^.

The nucleic acid bases have the characteristic aromatic properties of planarity and density of delocalized $\pi$ electrons^69^. In purines, the aromatic ring has ten delocalized $\pi$ electrons, while in pyrimidines, there are only six $\pi$ electrons^25^. In A-T and C-G junctions, the interplay between the intrinsic base–base terms through the H-bond produces an overlap of the $\pi$-cloud. The intermolecular H atoms have a particular positive charge due to the attractive electron force exerted by the electronegative substituents with N or Oxygen (O)^70^. Although all the H-bonds in the base pairs contribute equally to their approach^71^, only the central NH–N bond contributes to the aromatic ring. The $\pi$-$\pi$ alignment through the ${p}_{z}$ orbitals overlap between the N3 of pyrimidine and the N1 of the corresponding purine forms a new MO: $\Phi {\pi }_{AT}$ or ${\Phi \pi }_{CG}$ physically constituted by the linear combination of $\pi$-MO from A (${\Phi \pi }_{A}$) and T (${\Phi \pi }_{T}$) or C (${\Phi \pi }_{C}$), and G (${\Phi \pi }_{G}$), respectively. Thus, we have two coherent quantum states that become one because the delocalized electrons of the $\pi$ cloud now move from T to A and from C to G, and vice versa:

$$\Phi {\pi }_{AT}= \sum_{i=1}^{n}\alpha_{i}\phi_{i} = \alpha_{1}{\phi }_{1}+ \alpha_{2}{\phi }_{2}\text{with }{\phi }_{1}={\Phi }_{\pi A} \text{and }{\phi }_{2}={\Phi }_{\pi T}$$

$${\Phi \pi }_{CG}= \sum_{i=1}^{n}\alpha_{i}\phi_{i}= \alpha_{1}{\phi}_{1} + \alpha_{2}{\phi}_{2},\text{with }{\phi}_{1}={\Phi }_{\pi C}{\phi}_{2}={\Phi }_{\pi G}$$

with $$n=2$$.

Unique non-covalent stacking $\pi$-$\pi$ interaction effects associated with delocalized electrons confer unique properties to the composite system, such as its low reactivity and high stability.

Making an analogy with a coordination complex, in A-T and C-G base pairs, the coordinated internal sphere has a nucleus formed by the central NH–N bond surrounded by the new $\pi$-MO (${\pi }_{AT}$ or ${\pi }_{CG}$). The eight electron pairs are equivalent to clouds of negative charge that are directed from near the central H atom toward the corners of the complex. It forms an electric field in a closed circular circuit. Also, because the linear combination of ${p}_{z}$ orbitals defines the $\pi$–$\pi$ interaction, the principal quantum number of the A-T or C-G coordination compound is $n=1$^72^. The ${p}_{z}$ orbitals overlap and electron–electron interactions tend to lead to specific regular geometries that maximize intra-electron group repulsions, being the electron pair arrangement linear for five electron groups and three lone pairs^4^. The structural organization of a complex is generally fixed and stable and is determined by the lowest possible energy arrangement^73^. This is equivalent to saying it adopts the most inferior internal stress arrangement.

## Nitrogenated bases pairs: Bose–Einstein Condensate analogue

The grouping of n quantum particles at the lowest energy level is called the Bose–Einstein Condensate (BEC)^74^. In the superconducting state, electrons form Cooper pairs^75^. The fermions interact via the exchange of phonons and can condense into a collective quantum state^76^. Also, the Cooper pairs are tied and have the same energy and phase. Analogous to BEC, the quantum condensate of Cooper pairs forming a superconductor takes place in a simple or fragmented form^77,78^. If the number of lattice sites (n) is one eigenvalue, the condensate is called simple, while it is called fragmented if more than one eigenvalue is of order n^78^.

In addition to Cooper pairs, condensation phenomena of different types of bosonic systems have been described, such as ultracold atoms^79^, magnons^79^, excitons^80,81^, and surface plasmons^82,83^. Some conditions must be met for the A-T or C-G system proposed here to be a candidate for BEC. The idea is to consider the atoms as fixed points in the crystal lattice with a positive charge and represent the free electrons as a homogeneous gas at a uniform potential^84^. In a BEC, a collective and coherent oscillation of free electron pairs exists^85^. Atoms share a common quantum state. All particles share the same wave function phase, allowing them to act as coherent waves. Some notable properties include quantum interference patterns and superfluidity^86^.

From the BEC model, the in-phase ${p}_{z}$ overlap gives the couplings between A-T and C-G coordination complexes. It results in constructive interference and produces a new orbital extended in the base pair's plane with a longer wavelength and lower energy. Orbitals with similar energies will have the most robust interactions and form a state of quantum coherence. The delocalized electrons occupy the lowest energy level, forming an oscillatory resonant quantum state between electron and hole pairs in the nitrogenous bases. Studies have shown that the ground state in DNA is strongly destabilized by the loss of $\pi$-bond stabilization^87–89^.

To be a BEC, the total momentum with the electronic coupling between the molecular components in the base pairs should be reduced to zero (zero momentum state P = 0)^86^. First, the positive $pp\sigma$, and negative $pp\pi$, interaction between two interacting atomic *P*_*z*_ orbitals can cancel each other, leading to a small net atomic pair interaction. Second, some rather large, predominantly $s$ and $p$ base interactions can cancel each other when added up to calculate the total base pair coupling. In A-T and C-G systems, the rings are composed of nine Carbons and six N joined by an H atom (if the significant contribution for the interaction corresponds to nitrogenous bases, functional groups were not included). Each Carbons has six protons, six neutrons, and six electrons, four of which are the valence electrons of the $\sigma$-bonding. Each N has seven protons, seven neutrons, and seven electrons, five of which are of valence. The system comprises 56 protons, 56 neutrons, and 56 electrons. The H atom only has one proton and one electron. Thus, the system condenses because the number of particles gives a total spin of zero, like in ^4^He_2_^90^ (Table 1).

**Table 1:** Zero momentum state (P = 0) in A-T and C-G base pairs condensates.

Base pair	Carbon	Nitrogen	H +
Atoms numbers	9	6	1
Adenine–thymine	56 e^−^	42 e^−^	1 e^−^ 1 p^+^
	56 n	42 n	
	56 p^+^	42 p^+^	
Atoms numbers	9	6	1
Guanine-cytosine	56 e^−^	42 e^−^	1 e^−^ 1 p^+^
	56 n	42 n	
	56 p^+^	42 p^+^	

The pair interaction cannot break the resonant states in A-T and C-G systems, so we consider it weak. In bosonic systems based on fermions, it must be assumed that the weak interaction allows the system to condense, and they all occupy a single energy level. This means the system's wavefunction decomposes into a product of identical single-particle wavefunctions. The ground state is entirely described by a single particle wavefunction $\psi$^91^. Therefore, in aromatic compounds, the arrangement of an annular current is present in the electronic structure of the ground state wave function^92,93^.

## Nitrogenated bases A, T, C, and G: a superconductor state

The fundamental difference between superconductivity and normal metallic conduction lies in the presence of electron pairs (Cooper pairs) in the former, while in the latter, the electrons move independently. Superconductors form a particular group of materials with high electric conductivity^94^. The microscopic model of superconductivity elaborated by the BCS Theory explains how the electron waves in the superconducting state don’t act independently, as in the Bloch model^45^. Therefore, considerable overlap exists between the wave functions of the individual Cooper pairs acting as a unit, resulting in a strong correlation between the pairs' movements^95^. When the Fermi surface lies within a single conduction band, for example, say at $n=1$, it can be argued that it is justified to "project" the multi-band Hamiltonian into an effective single-band model. Suppose that the interaction between bands is weak and, at the same time, all bands except the $n=1$ are far from the Fermi surface. Since no scattering or inelastic collisions exist, the resistance disappears, and the material becomes superconducting^96^.

Classical superconductivity depends on temperature and pressure^96^. The Drude-Lorentz model introduced the idea that electrical resistance is due to collisions of electrons with impurities and imperfections, especially with the lattice vibrations of the crystal^97^. The lattice vibrations will decrease with the temperature reduction because entropy, representing disorder, also declines^98^. The resistance of some materials suddenly drops to zero below a specific temperature, called the critical Temperature ${(T}_{c})$^95,99^. They become superconducting, meaning they can conduct currents without energy loss.

Although the BCS pairing Theory through the electron–phonon interaction mechanism is generally believed to describe the superconductivity of ‘conventional’ superconductors, both type I and II, in recent years, an increasing number of materials failed to be explained by it^100^ Various experimental observations point to a particular role of charge asymmetry and the electric current produced between holes in superconducting states^101,102^. Neither BCS Theory nor London, Ginzburg–Landau, and Abrikósov's Theories include it in the foundation of superconductivity. Since the breakthrough discovery of chemically doped polyacetylene possessing electrical conductivity, $\pi$-conjugated polymers have been of great interest^103^. In doped $\pi$-conjugated polymers, one type of charge carrier, either holes or electrons, predominates^104^. Doping reduces the energy level band gap between the LUMO and the HOMO with an increase in conductivity^102^.

Here, we describe a superconductivity model that depends on the oscillatory resonant movement of correlated electron and hole pairs in a single band ($\pi$-MO) without considering the temperature or pressure as a critical parameter. The clouds of negative charge from 16 electrons and hole pairs exist in the nitrogenous bases A, T, C, and G with a finite momentum P, creating a state with a spatially modulated Cooper-pair density. The electron pairs move to occupy the hole pairs around the Fermi level in the conduction band, corresponding to our model's $\pi$-MO energy level. Simultaneously, the displacement of other electron pairs occurs, occupying the holes left by the previous one. The electric field is confined in $\pi$-$M{O}_{T},$ $\pi$-$M{O}_{A}$, $\pi$-$M{O}_{C}$, and $\pi$-$M{O}_{G}$ containing the electron and hole pair density wave state. The electron cloud can no longer be deflected or interact with others. Thus, the oscillatory resonant state between the electron and hole pairs generates a single-band supercurrent in the structure that occurs infinitely in time. The continuous electrical current without resistance converts A, T, C, and G in superconductors. We call this state the static superconductivity of the pairs oscillating resonantly. A damped oscillator with resonant interaction always keeps its energy finite.

The current density for $n$ charges $q$ moving with a velocity $v$ across a surface $s$ is: $\mathcal{J}=\frac{\text{I}}{\mathcal{S}}$ or $\mathcal{J}=nqv$ with $n$ as the charge concentration.

If the medium is continuous as a conductor, then $\mathcal{J}=\rho \upsilon$ with $\rho =$ charge density.

From the quantum point of view, $\rho ={\left|\psi \right|}^{2}$. The current density is $\mathcal{J}=\frac{\hslash }{2{m}_{i}}\left({\psi }^{*}\nabla \psi -\psi \nabla {\psi }^{*}\right)$ where: $\hslash =\frac{h}{2\pi }$.

For correlated pairs, the current density of a single pair is $\mathcal{J}=\left(2e\right)\nu$, and for the collective, is $\mathcal{J}=n\left(2e\right)\nu$, with $\nu =linear velocity$. It needs a magnetic and an electric field. In DNA, the pairs are confined into a single molecular orbital traveling in a circular path through the entire molecule. Thus, $v=rw$ where $r$ is the radius of $\pi$-MO and $w$ is the angular velocity of the pairs.

Finally, $\mathcal{J}=n\left(2e\right)rw=\rho rw$, with $\mathcal{J}=\rho \upsilon$ and $\rho ={\left|\psi \right|}^{2}$, the current of pairs would be $\text{I}=\int j\mathcal{d}S$.

## A-T and C-G: Josephson Effect between two superconductors

The Josephson Junction is one of the bases of quantum communication and quantum computing. It refers to any insulator material sandwiched between two superconductors^105^. An electric current manifests the Josephson Effect due to the Tunnel Effect between two superconductors separated by a thin insulator without external bias voltage^106^. The superposition of the wave functions of the superconductors causes the current through the Josephson Junction to depend sinusoidally on the phase difference^74^. An atomic-scale 0−$\pi$ transition in a Josephson Junction has been described^107,108^.

The H-bond enhances the chemical stability, geometry, and reactivity of the coordination sphere within some molecular compounds^73,109^. When the A-T or C-G composite systems enter the Josephson regime, the weak link is expected to act as a nonlinear inductive element because the Josephson current, a nonlinear function of the quantum phase, gives the current through it. The delocalized electrons are moving together in a collective movement as a gas of free electrons confined in $\pi$-$M{O}_{AT}$ or $\pi$-$M{O}_{CG}$ around the central NH–N. The condition defined by Cooper is established: one electron slightly displaces a partially positively charged H atomic nuclei towards itself as it passes due to Coulomb attraction. Electron number two "sees" a region with a higher positive charge density relative to the surroundings and is therefore attracted to this region and, thus, indirectly to electron one^110^. Due to the exclusion principle, the two electrons in a pair Cooper must have opposite spin, as is true in our model.

In the A-T junction, the N3 of T has a lone pair of electrons that is part of its $\pi$ cloud. In A, the N1 gives an electron to its $\pi$ cloud and uses the lone pair to attract the H attached to the N3 of T. This system forms the A-T Josephson Junction that allows the correlation of electrons from the $\pi$-$M{O}_{T}$ with electrons from the $\pi$-$M{O}_{A}$ by a resonant effect. Similarly, in C-G, the N3 in C uses its lone pair of electrons to form an H-bond with the N1 of G. This system forms the C-G Josephson Junction. Using the typical structure X − H···Y, where X is the donor atom, and Y is the acceptor, the X − H distance has been calculated at ≈110 pm, whereas the H···Y distance is between 160 and 200 $\text{pm}$^111^. The coherence length (coherence length, $\xi$) in a typical Josephson Junction is about 10 μm^94^.

The current across the Josephson junction in two BEC superconductors, A-T or C-G, with gap $\Delta$ and phases ${\varnothing }_{A}$, ${\varnothing }_{T}$ or ${\varnothing }_{C}$, ${\varnothing }_{G}$:

Using the equation in Sukhatme et al.^90^ .

$$\text{I}\left(\phi \right)={\text{I}}_{j}\text{sin}{\varnothing }_{AT} or {\varnothing }_{CG},$$

where $${\varnothing }_{AT}={\varnothing }_{A}-{\varnothing }_{T}$$ or $${\varnothing }_{CG}={\varnothing }_{C}-{\varnothing }_{G}$$

${\varnothing }_{AT} or {\varnothing }_{CG}$ is the quantum phase difference across the junction. According to Levy et al.^74^ , the phase difference evolves:

$$\varnothing =-\frac{\Delta \mu }{\hslash }$$

The gap $\Delta$ is the binding energy of the new electron pairs that cross the junction.

${\text{I}}_{j}$: is the junction critical current, and it is represented by:

$${\text{I}}_{j}{R}_{N}= \frac{\pi \Delta }{2e} taugh \left(\Delta /2{K}_{B}T\right)$$

where ${R}_{N}$ is the resistance of the normal state of the junction.

${\text{I}}_{j}{R}_{N}=V$: by Ohm's Law is the potential difference of the junction.

${K}_{B}T=Q$: the thermal energy of the junction.

$2e$: charge of the correlated electron pair.

**Figure 4:**
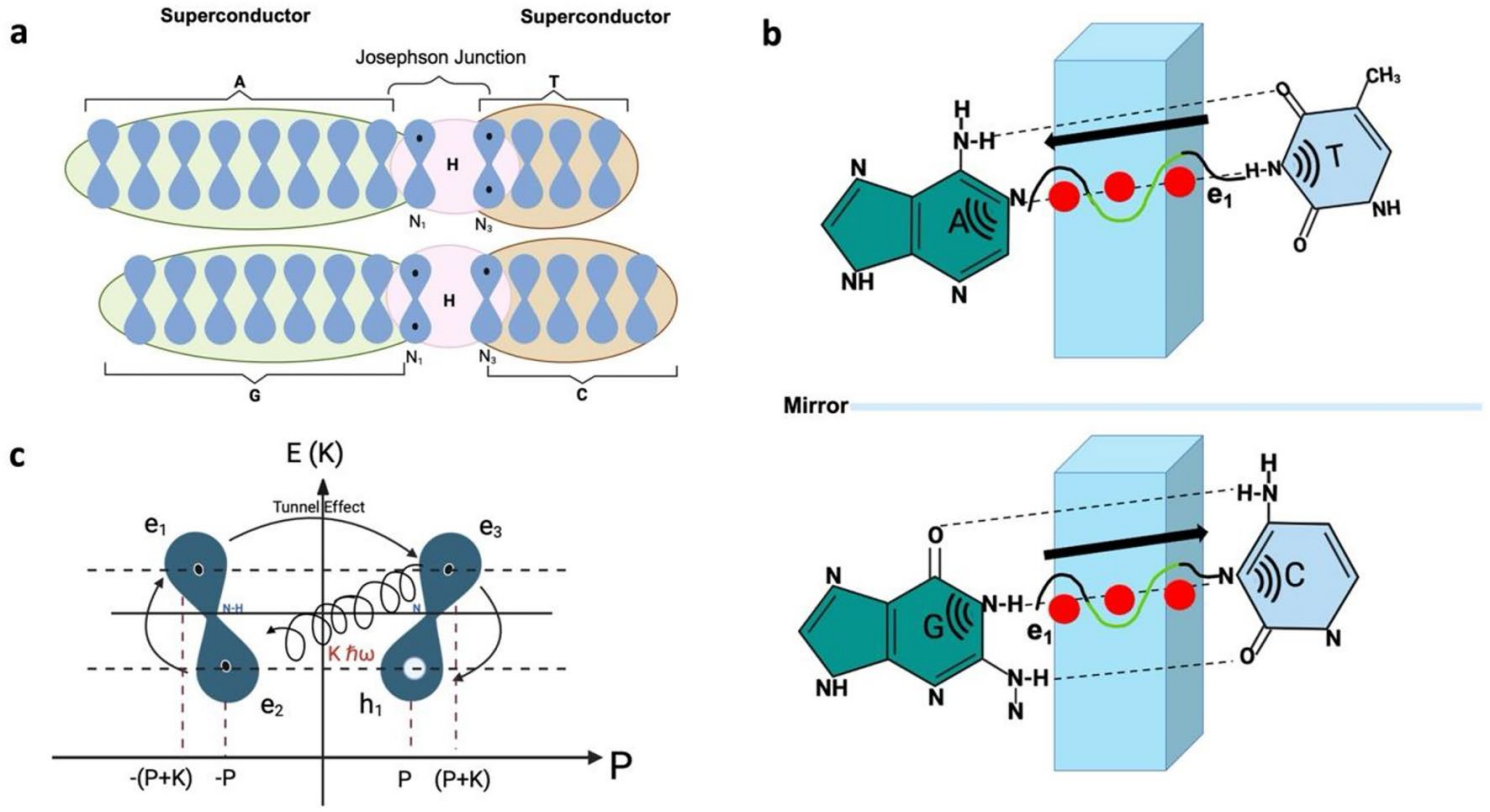
Josephson Effect in A-T and C-G coordination complexes. (**A**) The central H-bond (NH–N), connecting two canonical nitrogenous bases, constitutes the Josephson junction. (**B**) Tunnel Effect: ${e}_{1}$ crosses the Junction without losing energy and with the same momentum but the opposite sign. (**C**) Oscillatory resonant quantum states between electron and hole pairs in N_1_ and N_3_ in-face ${p}_{z}$ orbitals.

Previously, we analyzed the formation of correlated electron and hole pairs in A, T, C, and G. Now, we will analyze the Josephson Effect in A-T and C-G coordination complexes. The formation of pairs must always meet two conditions: 1-There must be electrons and holes in the interacting orbitals, and 2-The energy difference of the electrons must be equal to the quantized molecular lattice vibrations. Electrons have opposite momentum $\pm K$. ${e}_{3}$ with the momentum $P+K$ emits a ${B}_{b}=\hslash \omega$ a ${e}_{2}$, losing the momentum $K$ and occupying the position of ${h}_{1}$ with momentum $P$. Then, ${e}_{1},$ through the Tunnel Effect, passes through the barrier (NH–N) to the position of ${e}_{3}$, where there is now a hole. ${e}_{1}$ conserved its energy, and the value of the momentum $-\left(P+K\right)$, only changed its direction to $P+K$ (Fig. 4).

In contrast to the individual nucleobases, in A-T and C-G, the attractive force is ${B}_{b}=\hslash \omega$, where $\omega$ is the frequency of the Resonance-Assisted H-bond (RAHB). It is a strong H-bond type^112,113^ characterized by the $\pi$-delocalization that involves H and cannot be described adequately by the electrostatic model alone^114^. Gilli and coworkers first proposed that two ends of a $\pi$-conjugated system were associated by an intramolecular H-bond interaction, which shortens the distance between them^115^. Such systems have amplified cooperation between the π-electron delocalization and the H-bond.

## A-T and C-G coordination complexes: magnetic properties

The magnetic properties generally depend on the complex's number of unpaired electrons^116^. If one or more unpaired electrons exist, the complex is paramagnetic and attracts magnetic fields proportional to the number of unpaired electrons. Without unpaired electrons, the compound will be diamagnetic and slightly repelled by magnetic fields^117^. In addition to zero electrical resistance, superconductors also have perfect diamagnetism. In a quantum mechanical system, the electrons will flow around the ring’s perimeter in the requisite direction only if all required ionic situations can mix and mediate a continuous flow that generates the diamagnetic ring current^118^. The current direction generates a magnetic field that opposes the external field inside the ring. Thus, aromatic species, like Benzene, are expected to have diamagnetic robust effects since all possible ionic structures assist the electron flow^54,119^.

In A nitrogenous base:

There is a current that goes from N1 to N9, characterized by the current density vector $\overrightarrow{{J}_{A}}$, and with a magnetic field $\overrightarrow{{B}_{A}}$ characterized by the Ampere equation:

$$\nabla \times \overrightarrow{{B}_{A}}=\frac{4\pi }{C} \overrightarrow{{J}_{A}},$$

where *c* is the speed of light.

In T nitrogenous base:

There is a current that goes from N1 to Carbon six, characterized by the current density vector $\overrightarrow{{J}_{T}}$, and with a magnetic field $\overrightarrow{{B}_{T}}$ characterized by the Ampere equation:

$$\nabla \times \overrightarrow{{B}_{T}}=\frac{4\pi }{C} \overrightarrow{{J}_{T}}$$

**Figure 5:**
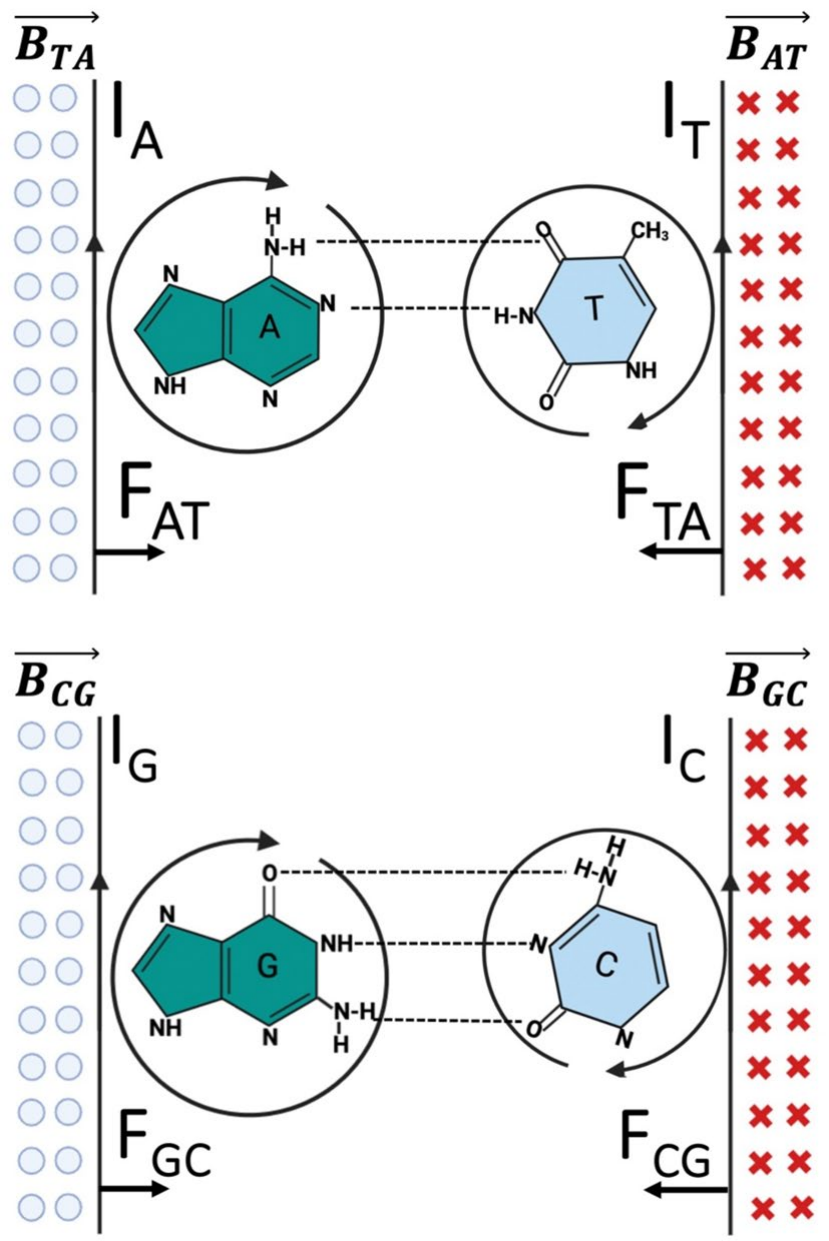
Magnetic field in A-T and G-C coordination complexes. A continuous flow that generates the diamagnetic ring current since all possible ionic structures assist the electron flow. I_G_ (I_A_) current generates a magnetic field in the conductor with I_C_ (I_T_) current, represented by crosses that are lines of field forces entering the plane and producing the force F_CG_ (F_TA_) in the conductor I_C_ (I_T_)_._ On the other hand, the conductor with current I_C_ (I_T_) creates a magnetic field represented by balls, which means that the lines of forces leave the plane, creating the force F_GC_ (F_AT_) in the conductor with current I_G_ (I_A_).

Both currents ${\text{I}}_{A}=\int \overrightarrow{{J}_{A}}\overrightarrow{. ds}$ and ${\text{I}}_{T}=\int \overrightarrow{{J}_{T}.}\overrightarrow{ ds}$ has the same direction and sense. Then, the magnetic field $\overrightarrow{{B}_{A}}$ exerts an attractive magnetic force on the conductor with current ${\text{I}}_{T}$, and vice versa, resulting in an attractive force between the nitrogenous bases; otherwise, A and T bases do not bind or attract to form a coordination complex (Fig. 5).

$\overrightarrow{{B}_{A}}$ and $\overrightarrow{{B}_{T}}$ have different values but the same direction, producing a magnetic field at the junction $\overrightarrow{{B}_{AT}}=$ $\overrightarrow{{B}_{A}} + \overrightarrow{{B}_{T}}$

The same occurs in the C-G coordination complex.

Magnetic susceptibility exaltations are closely associated with aromaticity^120^. In A-T and C-G, the magnetic effects are related to the intrinsic property of the $\pi$ system to sustain a circular $\pi$ flow due to the presence of oscillating correlated electron and hole pairs in resonant quantum states. Spherical parentages have been calculated based on the Huckel eigenvectors^121^. The ring current contribution to a cyclic system's total magnetic susceptibility is proportional to the product of the square of the ring area and the aromatic stabilization energies^120^.

## Symmetries of DNA code and quantum informational cryptography

DNA contains only two essential Watson–Crick base pairs, but there are ten independent base pair steps (two consecutive base pairs along the double helix). The double-stranded base pair complementarity has one geometrical and one electronic component. The strong H-bonded complexes are possible because of the electronic component's stability inside the molecule's geometrical configuration^114^. For example, the self-dimerization of A-A, U-U, and A-U is possible by two H-bonds, but the A-U dimer is more stable with a higher association constant^122^. Human telomeric sequences have a hybrid-type intramolecular G-quadruplex structure due to the charge separation that goes with donor–acceptor orbital interactions and not from the strengthening caused by resonance in the $\pi$ electron system. In DNA G-quadruplexes' intriguing cooperativity, the $\pi$ delocalization provides only an extra stabilization to the H-bonds^123^.

**Figure 6:**
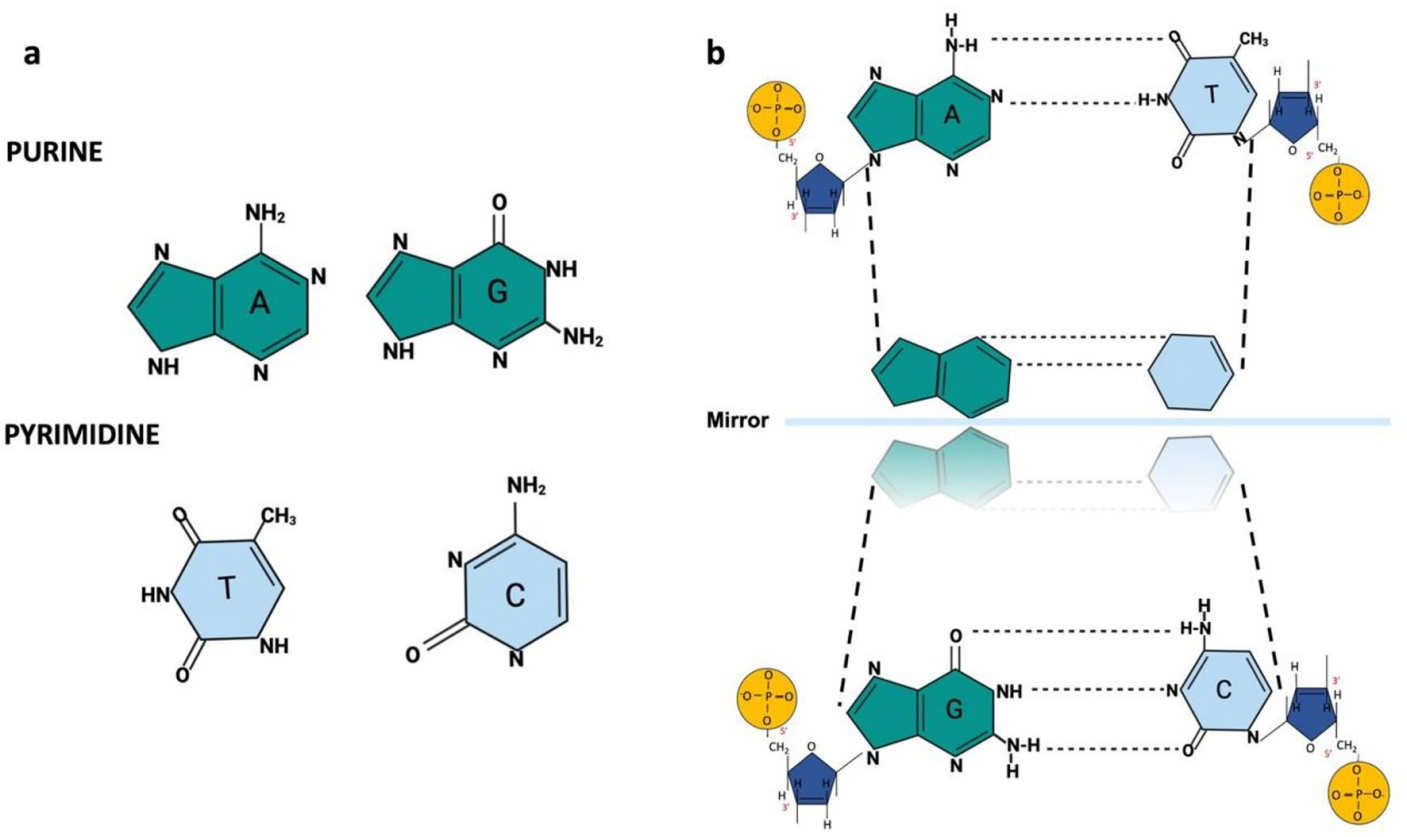
Symmetries of DNA canonical base pairs quantum informational cryptography. (**A**) Chemical structure of the nitrogenous bases, pyrimidines, and purines. (**B**) A-T and C-G, in their mutual connection, have the same functional quantum state while retaining different structures and morphologies.

If we examine A, T, C, and G individually, they are morphologically and chemically different. However, if we examine A-T and C-G in their mutual connection, the source of the self-motion that characterizes a single quantum state is discovered. Although A-T and C-G coordination complexes are structurally different, they are functionally similar systems (Fig. 6) Any bipartite state can be expressed in a pure state^11,124^. To State One, we will assign subsystems A and T sub-states for A-T and subsystems C and G sub-states for C-G pure state, respectively. A-T and C-G associations are highly specific while all other pairs of nucleotides are additive in their interactions. System Two will comprise the non-selective affinity between A, T, C, and G monomeric and hetero-derivatives (subsystems A-A, C–C, G-G, T-T, A-C, A-G, T-C, and T-G). System Two represents the degenerate states. In quantum mechanics, degeneracy is when the same energy level has more than one associated state^125^. For the Hamiltonian operator H_0,_ two of the eigenenergies have the same value, that is, there are two different eigenfunctions whose eigenvalues are the same^11,126,127^.

For a system of two qubits, there are two equivalence classes: the separable states and the entangled states. Based on the information above, we established the entangled states $|\psi \rangle X$ ($|\psi \rangle$ A-T and $|\psi \rangle$ C-G) fundamental states of the quantum system in the genetic code. The ground state of the quantum system represents its lowest possible energy state known as the zero-point energy of the system. In quantum mechanics, $E=\frac{\mathcal{h}\omega }{2}$ is associated with the ground state of the quantum harmonic oscillator $E=\hslash \omega \left(n+1/2\right)$ with $n=0$. More precisely, the zero-point energy is the expected value of the Hamiltonian of the system. The subsystems A-A, C-C, G-G, T-T, A-C, A-G, T-C, and T-G will constitute the degenerative states $|\psi \rangle AA, |\psi \rangle CC, |\psi \rangle GG, |\psi \rangle TT, |\psi \rangle AC, |\psi \rangle AG, |\psi \rangle TC$, and $|\psi \rangle TG$, respectively. They will be represented by $|\psi \rangle Y$.

As we mentioned, classical information can be converted entirely into quantum information, it is enough to classify it in orthogonal states. For example, in the case of qubits, it is enough to associate the state's $|0\rangle$, and $|1\rangle$, with their respective bits 0 and 1. In A-T and C-G, similar H-bond contributions have been determined^71^. In adittion, the A-T electronic angular frequencies have been calculated at 3.062, 2.822, and 4.24 in units of ${10}^{15}$ radians per second for ${\omega }_{xx},$ ${\omega }_{yy}$, and ${\omega }_{zz},$ respectively. In C-G, similar results were found: 3.027, 2.722, and 4.244 in units of ${10}^{15}$ radians per second for ${\omega }_{xx}$, ${\omega }_{yy}$, and ${\omega }_{zz}$^126^. In both sets of base pairs, the same functional quantum state, while retaining different structures and morphologies, has been demonstrated in this work. (Fig. 6)

$$\text{If }|\psi \rangle AT=|\psi \rangle CG=|\psi \rangle X,\text{ then }|0\rangle \left[\begin{array}{c}A\\ T\end{array}\right]=|0\rangle \left[\begin{array}{c}C\\ G\end{array}\right]=|0\rangle \left[X\right],\text{ and}$$

$$|\psi \rangle AA= |\psi \rangle CC= |\psi \rangle GG= |\psi \rangle TT= |\psi \rangle AC= |\psi \rangle AG=|\psi \rangle TC= |\psi \rangle TG=|\psi \rangle Y=|1\rangle \left[Y\right]$$

Separable states can be obtained deterministically from any other state: we must prepare each qubit in the state that corresponds to it^128^. Conversely, an interlocked state can only be obtained from another linked state. Maximally entangled states are significant because they can be obtained from any other state of two qubits deterministically^126^. The Bell States, or Einstein–Podolsky–Rosen (EPR) pairs in honor of the EPR paradox, form a database of maximally entangled states^128^. A natural question that comes to mind is that in addition to the existing two pairs of bases-entangled states, how does the codons' genetic information know the base pairs' quantum state?

## DNA: superdense coding for perfect teleportation

Protein-coding genes are made up of tri-nucleotide units called codons. There are 64 different codons in the genetic code. Three sequences, UAG, UGA, and UAA, are known as stop codons, and the sequence AUG, read as methionine, serves as the start codon. The DNA codons reading occur on the sense DNA strand and are arranged in a 5′-to-3′ direction. Every three consecutive nucleotides (the triplets are arranged linearly and continuously) act as a triplet combination that codes for an amino acid. Now we need to translate nucleotide triplets into the corresponding amino acid using the $$|0\rangle \left[\begin{array}{c}0\\ 1\end{array}\right]$$ and $$|1\rangle \left[\begin{array}{c}1\\ 0\end{array}\right]$$ qubits code and quantum mechanics.

Quantum teleportation is a prime example of a quantum information processing task where an unknown state can be perfectly transported from one place to another using previously shared entanglement and classical communication^17,18^. A multiparticle entangled state for quantum teleportation, like in the case of three qubits, is possible^129,130^. In addition, entangled states enhance classical information capacity^131^. In superdense coding, we can send the information of two classical bits stored in a qubit between two distant positions and through a quantum channel if they share an EPR pair^132–134^. More generally, if two positions share a maximally entangled state in the two-dimensional Hilbert space^9^, sending one qubit can communicate two log_2_d classical bits. Thus, a maximally entangled state doubles the classical information capacity of a channel if it belongs to one of the Bell basis states^135^.

Suppose that during transcription, the RNA polymerase teleports the quantum information of the codons using the combination of the maximally entangled base pairs A-T and C-G. Transcription stars when RNA polymerase binds to a promoter sequence near the beginning of a gen^136^. The region of opened-up DNA is called a transcription bubble^137^. The base pairs will be separated by a sufficient distance such that there can be no influence between both systems. Transcription uses one of the two exposed DNA strands as a template^136^. As A-T and C-G are maximally entangled states, they keep the same quantum state even when the pairs are separated. We have four possible combinations: A-T, T-A, G-C, and C-G.

$$|0\rangle \left[\begin{array}{c}A\\ T\end{array}\right]=|0\rangle \left[\begin{array}{c}T\\ A\end{array}\right]=|0\rangle \left[X\right],\text{ and }|1\rangle \left[\begin{array}{c}G\\ C\end{array}\right]=|1\rangle \left[\begin{array}{c}C\\ G\end{array}\right]=|1\rangle \left[Y\right]$$

Then, the RNA polymerase contacts only one element of the pair (classical information) in the template strand. Thus, we also must encode each nitrogenous base. Considering the number of aromatic rings, we can assign:

$$\text{Purines }=|0\rangle ,\text{Pyrimidines}=|1\rangle ,\text{ then}$$

$$|\psi \rangle A=|\psi \rangle G=|0\rangle ,\text{ and }|\psi \rangle T=|\psi \rangle C=|1\rangle ,\text{ then}$$

$$|0\rangle \left[X\right]=|0\rangle \left[01\right]\text{ or }|0\rangle \left[10\right],\text{ and }|1\rangle \left[Y\right]=|1\rangle \left[01\right]\text{ or} |1\rangle \left[10\right]$$

Combining the quantum state of the composite system with classical information, RNA polymerase teleports one of the four Bell states:

$$|\psi \rangle AT=00,|\psi \rangle TA=01,|\psi \rangle CG=11,\text{and }|\psi \rangle GC=10$$

The state of the A-T or C-G composite system is defined from their eigenstates as follows: $|\psi \rangle =\sum_{\mathfrak{i}}|\psi \rangle \mathfrak{i}$, where $|\psi \rangle \mathfrak{i}$ represents the different eigenstates of the system A-T or C-G, and these eigenstates are: $|\psi \rangle \mathfrak{i}=\sum_{\mathcal{i},\mathcal{j}}{C}_{\mathcal{i}\mathcal{j} }|\mathfrak{i}\rangle \otimes |\mathcal{j}\rangle$. The density operator $\rho =|\psi \rangle \langle \psi |$ can be represented as:

$$\rho AT (\rho CG) =\sum_{\begin{array}{c}i,j\\ {\mathcal{i}}^{,}{\mathcal{j}}^{,}\end{array}}{C}_{{\mathcal{i}}^{,}{\mathcal{j}}^{,}}^{\divideontimes }$$, where subsystem A or C is $|\mathcal{i}\rangle \langle {\mathcal{i}}^{,}|$ and subsystem C or G is $|\mathcal{j}\rangle \langle {\mathcal{j}}^{,}|$.

A two-dimensional vector can be created by adding multiples of the vectors (1,0) and (0,1): $$\left[\begin{array}{c}x\\ y\end{array}\right] =x\left[\begin{array}{c}1\\ 0\end{array}\right] +y\left[\begin{array}{c}0\\ 1\end{array}\right]$$. The most suitable base vectors are orthogonal, valid for (1,0) and (0,1). Two vectors are orthogonal if their dot product is zero, which means they are at 90 angles^138,139^. Similarly, two functions are orthogonal if their dot product is zero^138,140^.

## Simplest version of the teleportation protocol

We propose the following simple teleportation protocol to simulate a quantum computer based on the DNA qubits established in this work (the protocol is modified from^141^). We have two systems: the first comprises two spins ½ of A and T or two spins ½ of C and G, prepared in a known pure state $|\psi \rangle$. Also, we have the system U formed by a spin ½ in an unknown pure state $|\psi \rangle$. This U system would be the information we want to teleport to the Messenger RNA (mRNA) to “manufacture” the corresponding amino acid. Systems A and T or C and G cannot interact directly after the state is prepared since they are far apart (A and T or C and G will be spatially separated when the transcription bubble is formed).

Suppose the state $\left|\psi \rangle \right.X=$ $|AT\rangle$, $|CG\rangle$, $|TA\rangle$, or $|GC\rangle$ (depending on classical information) is:

$$\left|\psi \rangle \right.X=\left|singlet\rangle \right.=\frac{\left|\uparrow \rangle A\left|\downarrow \rangle T-\left|\downarrow \rangle A\left|\uparrow \rangle T\right.\right.\right.\right.}{\sqrt{2}}\text{ for A}-\text{T}, \left|\psi \rangle \right.X=\left|singlet\rangle \right.=\frac{\left|\uparrow \rangle C\left|\downarrow \rangle G-\left|\downarrow \rangle C\left|\uparrow \rangle G\right.\right.\right.\right.}{\sqrt{2}}\text{ for C}-\text{G},$$

$$\left|\psi \rangle \right.X=\left|singlet\rangle \right.=\frac{\left|\uparrow \rangle T\left|\downarrow \rangle A-\left|\downarrow \rangle T\left|\uparrow \rangle A\right.\right.\right.\right.}{\sqrt{2}}\text{ for T}-\text{A},\text{ or }\left|\psi \rangle \right.X=\left|singlet\rangle \right.=\frac{\left|\uparrow \rangle G\left|\downarrow \rangle C-\left|\downarrow \rangle G\left|\uparrow \rangle C\right.\right.\right.\right.}{\sqrt{2}}\text{ for G}-\text{C}$$

where $\left|\uparrow \rangle \text{S}\left|\downarrow \rangle \text{S}\right.\right.$ is the eigenstate of the operator ${S}_{Z}$ in the system $S$ (for $\text{S}=A, T, C, or G)$ with positive and negative projection. This state is an eigenstate of the total spin operator of the system ${J}^{2}={\left(\overrightarrow{A}+\overrightarrow{T}\right)}^{2},$ with eigenvalue 0, that is, the singlet state of the system. The state of the system $U$ is represented in the form $\left|U\rangle =\alpha \left|\uparrow \rangle \right.+\beta \left|\downarrow \rangle \right.\right.$, where $\alpha$ and $\beta$ are two unknown coefficients that satisfy: $\alpha *\alpha +\beta *\beta =1$. Thus, the initial state of the system ($\left|\psi \rangle \right.{X}_{i}$) is:

$$\left|\psi \rangle \right.{X}_{i}=\alpha \frac{\left|\uparrow \rangle A\left|\downarrow \rangle T\left|\uparrow \rangle U\right.-\left|\downarrow \rangle A\left|\uparrow \rangle T\right.\right.\left|\uparrow \rangle U\right.\right.\right.}{\sqrt{2}}+\beta \frac{\left|\uparrow \rangle A\left|\downarrow \rangle T\left|\downarrow \rangle U\right.-\left|\downarrow \rangle A\left|\uparrow \rangle T\right.\right.\left|\downarrow \rangle U\right.\right.\right.}{\sqrt{2}}\text{for A}-\text{T},$$

$$\left|\psi \rangle \right.{X}_{i}=\alpha \frac{\left|\uparrow \rangle C\left|\downarrow \rangle G\left|\uparrow \rangle U\right.-\left|\downarrow \rangle C\left|\uparrow \rangle G\right.\right.\left|\uparrow \rangle U\right.\right.\right.}{\sqrt{2}}+\beta \frac{\left|\uparrow \rangle C\left|\downarrow \rangle G\left|\downarrow \rangle U\right.-\left|\downarrow \rangle C\left|\uparrow \rangle G\right.\right.\left|\downarrow \rangle U\right.\right.\right.}{\sqrt{2}}\text{ for C}-\text{G},$$

$$\left|\psi \rangle \right.{X}_{i}=\alpha \frac{\left|\uparrow \rangle T\left|\downarrow \rangle A\left|\uparrow \rangle U\right.-\left|\downarrow \rangle T\left|\uparrow \rangle A\right.\right.\left|\uparrow \rangle U\right.\right.\right.}{\sqrt{2}}+\beta \frac{\left|\uparrow \rangle T\left|\downarrow \rangle A\left|\downarrow \rangle U\right.-\left|\downarrow \rangle T\left|\uparrow \rangle A\right.\right.\left|\downarrow \rangle U\right.\right.\right.}{\sqrt{2}}\text{for T}-\text{A},\text{ and}$$

$$\left|\psi \rangle \right.{X}_{i}=\alpha \frac{\left|\uparrow \rangle G\left|\downarrow \rangle C\left|\uparrow \rangle U\right.-\left|\downarrow \rangle G\left|\uparrow \rangle C\right.\right.\left|\uparrow \rangle U\right.\right.\right.}{\sqrt{2}}+\beta \frac{\left|\uparrow \rangle G\left|\downarrow \rangle C\left|\downarrow \rangle U\right.-\left|\downarrow \rangle G\left|\uparrow \rangle C\right.\right.\left|\downarrow \rangle U\right.\right.\right.}{\sqrt{2}}\text{for G}-\text{C}.$$

The interaction of type II restriction endonucleases with specific DNA sequences, causing quantum effects changes that result in the double-strand breakage, has been described^126^. Here, we suggest that the RNA polymerase, acting as a decoherence shield upon specific binding, creates decoherence-free subspaces for quantum entanglement. After RNA polymerase-specific binding, the Hamiltonian of the system is altered for a certain time t_1_ = $\frac{\pi }{\upsilon}$. For the base pair A-T, there is an interaction between spins at $T$ and $U$ with the Hamiltonian: ${H}_{TU}=b({S}_{x}^{\left(T\right)}-{S}_{Z}^{\left(U\right)}+\frac{\upsilon}{h}{S}_{x}^{\left(T\right)}{S}_{z}^{\left(U\right)})$. Where $b$ is a magnetic field of magnitude $\frac{\upsilon}{2}$ applied in the direction of the $X(Z)$ axis on the spin $T(U).$

Then, a biological mechanism acting like a CNot gate is probably applied. The operator $E=exp (-it_{1}{H}_{TU}/\hslash )={i}^{-1/2}{E}_{CNot}$ has the effect of multiplying the state by an irrelevant global phase ${i}^{-1/2}$, reversing the orientation of the spin at $T$ if the spin $U$ is $\left(\uparrow \right)$ and leave it in its original orientation if the spin $U$ is $\left(\downarrow \right)$.

For the pairs T-A, G-C, and C-G the Hamiltonian will be: ${H}_{AU}=b({S}_{x}^{\left(A\right)}-{S}_{Z}^{U}+\frac{\upsilon}{h}{S}_{x}^{A}{S}_{z}^{U})$, ${H}_{CU}=b({S}_{x}^{\left(C\right)}-{S}_{Z}^{\left(U\right)}+\frac{\upsilon}{h}{S}_{x}^{\left(C\right)}{S}_{z}^{\left(U\right)})$, and ${H}_{GU}=b({S}_{x}^{\left(G\right)}-{S}_{Z}^{\left(U\right)}+\frac{\upsilon}{h}{S}_{x}^{\left(G\right)}{S}_{z}^{\left(U\right)})$, respectively. The evolution operator will be for T-A, G-C, and C-G: $E=exp (-it_{1}{H}_{AU}/\hslash )={i}^{-1/2}{E}_{CNot}$, $E=exp (-it_{1}{H}_{CU}/\hslash )={i}^{-1/2}{E}_{CNot}$, and $E=exp (-it_{1}{H}_{GU}/\hslash )={i}^{-1/2}{E}_{CNot}$, respectively.

After this operation is applied, the system status will be:

$$\left|\psi \rangle \right.1={U}_{CNot}{\left|\psi \rangle \right.}_{i}=\alpha \frac{\left|\uparrow \rangle A\left|\uparrow \rangle T\left|\uparrow \rangle U\right.-\left|\downarrow \rangle A\left|\downarrow \rangle T\right.\right.\left|\uparrow \rangle U\right.\right.\right.}{\sqrt{2}}+\beta \frac{\left|\uparrow \rangle A\left|\downarrow \rangle T\left|\downarrow \rangle U\right.-\left|\downarrow \rangle A\left|\uparrow \rangle T\right.\right.\left|\downarrow \rangle U\right.\right.\right.}{\sqrt{2}}\text{ for A}-\text{T}$$

$$\left|\psi \rangle \right.1={U}_{CNot}{\left|\psi \rangle \right.}_{i}=\alpha \frac{\left|\uparrow \rangle C\left|\uparrow \rangle G\left|\uparrow \rangle U\right.-\left|\downarrow \rangle C\left|\downarrow \rangle G\right.\right.\left|\uparrow \rangle U\right.\right.\right.}{\sqrt{2}}+\beta \frac{\left|\uparrow \rangle C\left|\downarrow \rangle G\left|\downarrow \rangle U\right.-\left|\downarrow \rangle C\left|\uparrow \rangle G\right.\right.\left|\downarrow \rangle U\right.\right.\right.}{\sqrt{2}}\text{ for C}-\text{G}$$

$$\left|\psi \rangle \right.1={U}_{CNot}{\left|\psi \rangle \right.}_{i}=\alpha \frac{\left|\uparrow \rangle T\left|\uparrow \rangle A\left|\uparrow \rangle U\right.-\left|\downarrow \rangle T\left|\downarrow \rangle A\right.\right.\left|\uparrow \rangle U\right.\right.\right.}{\sqrt{2}}+\beta \frac{\left|\uparrow \rangle T\left|\downarrow \rangle A\left|\downarrow \rangle U\right.-\left|\downarrow \rangle T\left|\uparrow \rangle A\right.\right.\left|\downarrow \rangle U\right.\right.\right.}{\sqrt{2}}\text{ for T}-\text{A}$$

$$\left|\psi \rangle \right.1={U}_{CNot}{\left|\psi \rangle \right.}_{i}=\alpha \frac{\left|\uparrow \rangle G\left|\uparrow \rangle C\left|\uparrow \rangle U\right.-\left|\downarrow \rangle G\left|\downarrow \rangle C\right.\right.\left|\uparrow \rangle U\right.\right.\right.}{\sqrt{2}}+\beta \frac{\left|\uparrow \rangle G\left|\downarrow \rangle C\left|\downarrow \rangle U\right.-\left|\downarrow \rangle G\left|\uparrow \rangle C\right.\right.\left|\downarrow \rangle U\right.\right.\right.}{\sqrt{2}}\text{ for G}-\text{C}$$

Then, a magnetic field of intensity $b$ acts at U in the direction $\left(\text{1,0},1\right)/\sqrt{2}$, for a time ${T}_{H}=2\pi /b$:

${E}_{H}=i \text{exp}(-i{t}_{H}b ({S}_{x}^{\left(U\right)}+{S}_{z}^{\left(U\right)})/\sqrt{2}/\hslash =(1/\sqrt{2} \hslash )({S}_{x}^{\left(U\right)}+{S}_{z}^{\left(U\right)})$. It will produce the local unitary transformation. Applying this operation to $|\psi \rangle 1$:

$$\left|\psi \rangle 2={E}_{H}\right|\psi \rangle 2=\alpha \frac{\left|\uparrow \rangle A\left|\uparrow \rangle T\left|\downarrow \rangle U-\left|\downarrow \rangle A\left|\downarrow \rangle T\left|\downarrow \rangle U+\left|\uparrow \rangle A\left|\uparrow \rangle T\left|\uparrow \rangle U-\left|\downarrow \rangle A\left|\downarrow \rangle \right.T\left|\uparrow \rangle U\right.\right.\right.\right.\right.\right.\right.\right.\right.\right.\right.}{2}+\beta \frac{-\left|\uparrow \rangle A\left|\downarrow \rangle T\left|\downarrow \rangle U+\left|\downarrow \rangle A\left|\uparrow \rangle T\left|\downarrow \rangle U+\left|\uparrow \rangle A\left|\downarrow \rangle T\left|\uparrow \rangle U-\left|\downarrow \rangle A\left|\uparrow \rangle \right.T\left|\uparrow \rangle U\right.\right.\right.\right.\right.\right.\right.\right.\right.\right.\right.}{2}=\frac{1}{2}\left[\left(\alpha \left|\uparrow \rangle +\beta \left|\downarrow \rangle )A\left|\uparrow \rangle T\left|\downarrow \rangle U-(\alpha \left|\downarrow \rangle -\beta \left|\uparrow \rangle \right.\right.\right.\right.\right.\right.)A\left|\downarrow \rangle T\right.\left|\uparrow \rangle \right.U+\alpha \left|\uparrow \rangle -\beta \right.\left|\downarrow \rangle \right.\right)A\left|\uparrow \rangle T\left|\uparrow \rangle U-\right.\right.(\alpha \left|\downarrow \rangle \right.+\beta \left|\uparrow \rangle )A\left|\downarrow \rangle T\left|\downarrow \rangle U\right.\right.\right.\right]\text{for A}-\text{T},$$

$$=\alpha \frac{\left|\uparrow \rangle C\left|\uparrow \rangle G\left|\downarrow \rangle U-\left|\downarrow \rangle C\left|\downarrow \rangle G\left|\downarrow \rangle U+\left|\uparrow \rangle C\left|\uparrow \rangle G\left|\uparrow \rangle U-\left|\downarrow \rangle C\left|\downarrow \rangle \right.G\left|\uparrow \rangle U\right.\right.\right.\right.\right.\right.\right.\right.\right.\right.\right.}{2}+\beta \frac{-\left|\uparrow \rangle C\left|\downarrow \rangle G\left|\downarrow \rangle U+\left|\downarrow \rangle C\left|\uparrow \rangle G\left|\downarrow \rangle U+\left|\uparrow \rangle C\left|\downarrow \rangle G\left|\uparrow \rangle U-\left|\downarrow \rangle C\left|\uparrow \rangle \right.G\left|\uparrow \rangle U\right.\right.\right.\right.\right.\right.\right.\right.\right.\right.\right.}{2}$$

$$=\frac{1}{2}\left[\left(\alpha \left|\uparrow \rangle +\beta \left|\downarrow \rangle )C\left|\uparrow \rangle G\left|\downarrow \rangle U-(\alpha \left|\downarrow \rangle -\beta \left|\uparrow \rangle \right.\right.\right.\right.\right.\right.)C\left|\downarrow \rangle G\right.\left|\uparrow \rangle \right.U+\alpha \left|\uparrow \rangle -\beta \right.\left|\downarrow \rangle \right.\right)C\left|\uparrow \rangle G\left|\uparrow \rangle U-\right.\right.(\alpha \left|\downarrow \rangle \right.+\beta \left|\uparrow \rangle )C\left|\downarrow \rangle G\left|\downarrow \rangle U\right.\right.\right.\right]\text{ for C}-\text{G},$$

$$=\alpha \frac{\left|\uparrow \rangle T\left|\uparrow \rangle A\left|\downarrow \rangle U-\left|\downarrow \rangle T\left|\downarrow \rangle A\left|\downarrow \rangle U+\left|\uparrow \rangle T\left|\uparrow \rangle A\left|\uparrow \rangle U-\left|\downarrow \rangle T\left|\downarrow \rangle \right.A\left|\uparrow \rangle U\right.\right.\right.\right.\right.\right.\right.\right.\right.\right.\right.}{2}+\beta \frac{-\left|\uparrow \rangle T\left|\downarrow \rangle A\left|\downarrow \rangle U+\left|\downarrow \rangle T\left|\uparrow \rangle A\left|\downarrow \rangle U+\left|\uparrow \rangle T\left|\downarrow \rangle A\left|\uparrow \rangle U-\left|\downarrow \rangle T\left|\uparrow \rangle \right.A\left|\uparrow \rangle U\right.\right.\right.\right.\right.\right.\right.\right.\right.\right.\right.}{2}$$

$$=\frac{1}{2}\left[\left(\alpha \left|\uparrow \rangle +\beta \left|\downarrow \rangle )T\left|\uparrow \rangle A\left|\downarrow \rangle U-(\alpha \left|\downarrow \rangle -\beta \left|\uparrow \rangle \right.\right.\right.\right.\right.\right.)T\left|\downarrow \rangle A\right.\left|\uparrow \rangle \right.U+\alpha \left|\uparrow \rangle -\beta \right.\left|\downarrow \rangle \right.\right)T\left|\uparrow \rangle A\left|\uparrow \rangle U-\right.\right.(\alpha \left|\downarrow \rangle \right.+\beta \left|\uparrow \rangle )T\left|\downarrow \rangle A\left|\downarrow \rangle U\right.\right.\right.\right]\text{for T}-\text{A},\text{ and}$$

$$=\alpha \frac{\left|\uparrow \rangle G\left|\uparrow \rangle C\left|\downarrow \rangle U-\left|\downarrow \rangle G\left|\downarrow \rangle C\left|\downarrow \rangle U+\left|\uparrow \rangle G\left|\uparrow \rangle C\left|\uparrow \rangle U-\left|\downarrow \rangle G\left|\downarrow \rangle \right.C\left|\uparrow \rangle U\right.\right.\right.\right.\right.\right.\right.\right.\right.\right.\right.}{2}+\beta \frac{-\left|\uparrow \rangle G\left|\downarrow \rangle C\left|\downarrow \rangle U+\left|\downarrow \rangle G\left|\uparrow \rangle C\left|\downarrow \rangle U+\left|\uparrow \rangle G\left|\downarrow \rangle C\left|\uparrow \rangle U-\left|\downarrow \rangle G\left|\uparrow \rangle \right.C\left|\uparrow \rangle U\right.\right.\right.\right.\right.\right.\right.\right.\right.\right.\right.}{2}$$

$$=\frac{1}{2}\left[\left(\alpha \left|\uparrow \rangle +\beta \left|\downarrow \rangle )G\left|\uparrow \rangle C\left|\downarrow \rangle U-(\alpha \left|\downarrow \rangle -\beta \left|\uparrow \rangle \right.\right.\right.\right.\right.\right.)G\left|\downarrow \rangle C\right.\left|\uparrow \rangle \right.U+\alpha \left|\uparrow \rangle -\beta \right.\left|\downarrow \rangle \right.\right)G\left|\uparrow \rangle C\left|\uparrow \rangle U-\right.\right.(\alpha \left|\downarrow \rangle \right.+\beta \left|\uparrow \rangle )G\left|\downarrow \rangle C\left|\downarrow \rangle U\right.\right.\right.\right]\text{for G}-\text{C}.$$

Now, if ${S}_{Z}$ is measured in T (A or C or G) and results that $T$($\downarrow$) (A $\left(\downarrow \right)$, or C $\left(\downarrow \right)$ or G $\left(\downarrow \right)$), then, $\pi$ rotation is performed around the X-axis of the complementary system A (T or G or C). If ${S}_{Z}$ is measured in T (A or C or G), but the result is $T$($\uparrow$) (A $\left(\uparrow \right)$ or C $\left(\uparrow \right)$ or G $\left(\uparrow \right)$), then, $\pi$ rotation is performed around the Z-axis of the complementary system A (T or G or C). The final state of the system $A+T+U$, or $T+A+U$, or $C+G+U$, or $G+C+U$ will be a product state, in which system is now in the initial $U$ state. If we wanted to teleport the state of a second system $\left|{U}^{*}\rangle \right.$, we need a new pair of spin (A-T, T-A, G-C, or C-G).

## Discussion

The amount of DNA data grows exponentially every year. The available technology cannot handle such volume necessitating the development of quantum computer accelerators in this area. Therefore, it is a topic of great interest for science to be able to find quantum systems of two states capable of functioning as a qubit. Any quantum system is in a state of thermal equilibrium and is related to the Hamiltonian of the system when the interactions with the environment are sufficiently weak. They must be stable against small perturbations from the outside. The equilibrium state of the system will be that which maximizes the entropy. The ground state is essential since it contains many qualitative characteristics of systems at low temperatures while being more easily tractable mathematically and computationally.

One of the main problems is that DNA's theoretical description is challenged by its intrinsic multiscale nature. In this work, to reproduce the properties of DNA, we described a model of aromaticity in the nitrogenous bases based on the formation of correlated electrons and hole pairs. Molecules have greater degrees of freedom, but in DNA, it is believed that the high mechanical tension associated with superhelicity limits the elastic response of DNA to distortion^142^. Considering the molecular crystal lattice's unique environment, we propose that the electron uses the energy from the quantized molecular vibration to create an oscillatory resonant quantum state between electron and hole pairs. According to the Electromagnetic Theory, two electrons will always repel themselves but must be drawn between them in an internal or external field influence. Here, the internal field is represented by ${B}_{b}=\hslash \omega$, where $\omega$ is the frequency of the individual nitrogenous base vibration or RAHB in the canonical base pairs. The direct relationship between the interaction energy and delocalization supports the importance of RAHB and $\pi$ systems^113,143^. Theoretically, the strength of the H-bond can be assessed using the non-covalent interactions index (NCI index), which allows visualization of these NCIs using the electron density of the system^144^.

The A-T base pair has two traditional H-bonds and one weak Carbon-H–O interaction^145^. H-bond cooperativity has been extensively studied in various compounds, including DNA^23^. Asensio and co-workers evaluated the difference between the sum of the individual H-bond energies and the total interaction of the base pairs in their regular coplanar geometries as the cooperative part of the H-bonding interactions^71^. In A-T, the sum of the magnitudes of the interaction energies for the individual H-bonds were 3.57 and 3.95 kcal/mol (second order MøllerPlesset (MP2) ab initio MO and Density Functional Theory (DFT) calculations) less than the interaction energy of the planar base pair. The cooperative contribution to the H-bond interaction was 31% of the total interaction for each molecular orbital method. The energetic analysis for the G-C base pair showed that the cooperative contributions for the three H-bond systems (3.05 and 4.32 kcal/mol) were of similar magnitude to that for the two H-bond systems A-T^71^. This similar fraction of cooperativity appears due to optimal geometries for each H-bond inside the two rigid molecules. This is relevant because differences between A-T and C-G qubits are established in some works based exclusively on the number of H-bonds^139^.

Since the electron-vibration coupling is weak, an additional interaction is needed to break the quantum resonant states. Then, a static current of correlated electron and hole pairs that is indefinite in time is generated around the perimeter of the ring. The electron-vibrational coupling and rich vibrational structures have been described previously^146–148^. For example, considering intramolecular vibrations of picene^-3^, the strength of electron–phonon coupling in K_3_picene was calculated^149^. Some studies have concluded that $\pi$ electrons, the more critical measure of aromaticity, decrease markedly with increasing ring size and vanish for systems with a diameter of around 1.3 nm^118,120^. This could be because the vibration interaction decreases with size. Moreover, in natural DNA monomers, the ultrafast and efficient transfer of the excited-state electronic energy into vibrational energy in the ground state has been demonstrated^142,150^. In addition, the ground state is strongly destabilized by the loss of aromaticity^87–89^.

BEC is a phenomenon in which macroscopic fractions of n particles accumulate in the same single-particle state. In contrast to a regular condensate, a fragmented condensate is strongly entangled, which leads to a simultaneous macroscopic occupation of several single-particle states^78^. Here, we propose a situation in which fragmentation arises from the interplay between interactions of electron and hole pairs in oscillatory quantum resonant estates. A-T and C-G systems are assemblies of spin-0, in which a $\pi$ bond has frozen the degrees of freedom of the electrons. A highly entangled state is constructed by assembling bosons by pairs of spin-zero. All atoms share the same spatial wave function, and only the spin state of each atom is relevant^151^. The collective spin is zero, too.

There are no collisions because RAHB is converted into the exchange energy between the pair’s correlational electrons and holes in the oscillatory quantum resonant states. The kinetic energy of the atoms plays a negligible role because the collision rate is small relative to the BCE, and there is no broadening due to spatial or time variations of the magnetic field. Thus, the A-T and C-G systems function like a superconductor. In this case, the coordination complex may constitute a superconductor condensate consisting of two atomic and molecular components connected through the intercomponent Josephson coupling. Various superconductors are characterized by the spin-up and spin-down pairing of the electrons that constitute the superconducting flow^92^. The spin pairing phase between two weakly interacting superconductors having Cooper-pair spin configurations $\left|\uparrow \rangle \right.$, and $\left|\downarrow \rangle \right.$, make the A-T and C-G system like a couple with two energy levels. That is, they behave like a quantum bit. These qubits are robust to decoherence and function as topologically protected quantum memories. Superconducting qubits are a promising technology for so-called topological quantum computing^7,12,15^.

London first described the relationship between aromaticity and superconductivity in 1937^152^. Studies have shown superconductivity and its enhancement in polycyclic aromatic hydrocarbons^92,93^. In the last few decades, it has been almost generally accepted that aromaticity is associated with the ground-state properties of cyclic $\pi$ electron compounds, and a $\pi$ electron ring current is induced when the system is exposed to external magnetic fields^93^. However, other authors proposed the existence of a spin current in the ground state of such molecules in the absence of an applied field^92^. Thus, superconductivity also describes the ground state of aromatic molecules. The momentum vectors of the up-and-down spin of electrons and holes in various aromatic hydrocarbons were also analyzed^153^. VB Theory calculations predict that the $\pi$ electrons in aromatic rings are localized and move in a one-dimensional half-filled band, and the aromatic character results from the coupling of electron spin^93^.

**Figure 7:**
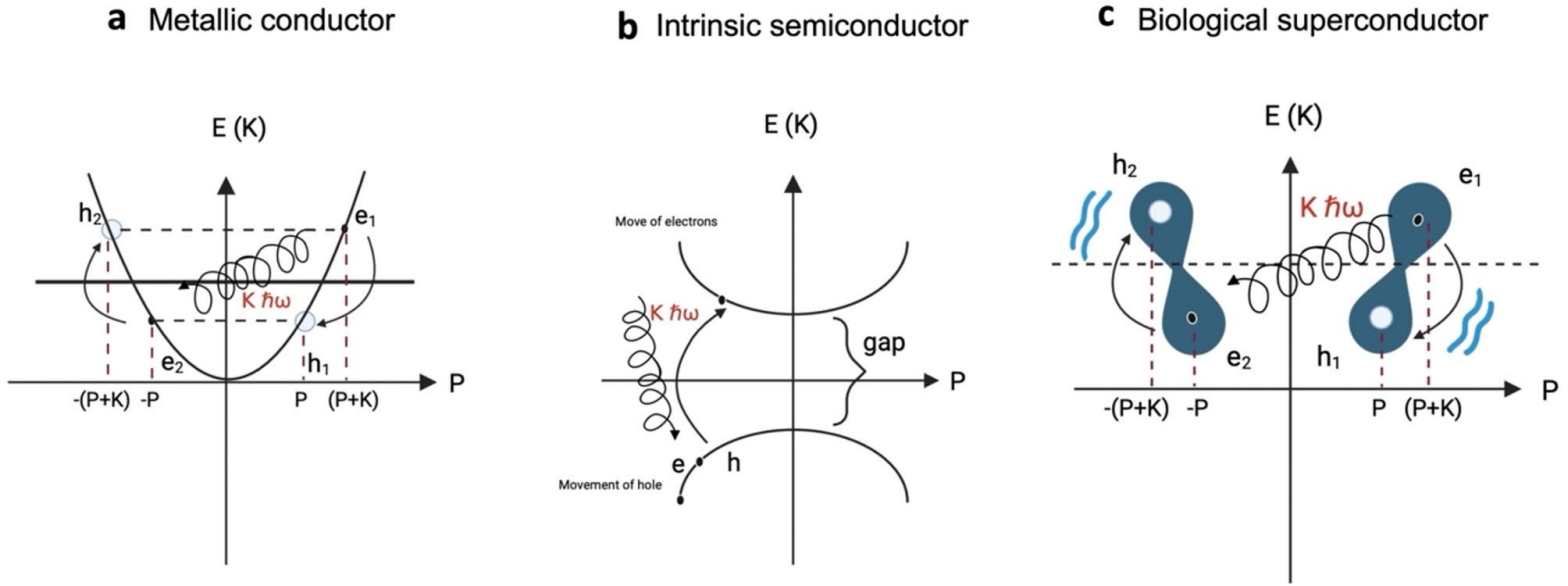
The electric current is presented in a metallic solid, a semiconductor, and DNA. (**A**) Correlated electron and hole pairs in a metallic conductor upon reaching the ${T}_{C}$. (**B**) In a semiconductor, there is no pair formation. (**C**) Oscillatory resonant quantum state between electron and hole pairs in DNA nucleobases.

Hybrid solids formed by the interaction between two individual molecular networks combined in the same crystal lattice with two or more physical properties have been designed. Magnetic molecular conductors like the salt [TTF]^δ+^[TCNQ]^δ-^ are a pack of donor and acceptor molecules to give delocalized electron energy bands due to overlap between the $\pi$ orbitals of adjacent molecules^100,154^. Electron delocalization is also found in molecular superconductors formed by cation-radical salts of organic donors, formulated as [donor]_m_X_n_^154,155^. The electronic interactions between $\pi$ and $d$ electrons orbitals could significantly stabilize superconductivity in molecular-based magnetic materials^156^. In general, due to their rich $\pi$ excessive nature, conjugated systems consisting of alternating single and double bonds with extensive $\pi$ conjugated backbones and delocalized electronic structures are considered an advanced class of materials in electronics^69^. (Fig. 7) Another remarkable finding is that *p* orbitals are believed to be significant in superconductors. For example, O $2p$ orbitals are in high-T oxides, Carbon $2p$ orbitals are in fullerenes, and the metallic conductivity of several substances^56^.

This work proposes a deep connection between aromaticity and superconductivity in DNA base pairs due to the formation of oscillatory resonant quantum states between correlated electrons and hole pairs. Eley and Spivey first envisioned DNA with a possible conducting behavior via the $\pi$-$\pi$ interaction of the stacked nucleobases^157^. In the 1990s, numerous studies described the intriguing electronic properties of DNA^158–160^. Also, it has been proposed to use DNA as a dielectric material in field-effect transistors and organic light-emitting diodes^161,162^. Kasumov et al. even induced superconductivity in DNA^163^. However, experimental outcomes in other works are amazingly different, covering DNA results as an insulator^164^. Remarkably, in all these works, the conduction in DNA has tested the hole or electron migration, considering sequence-specific effects in neighboring base pairs. Suppose the coupling between adjacent base pairs is strong enough. In that case, ${p}_{z}$ orbitals perpendicular to the base plane overlap sufficiently to form delocalized $\pi$ bonding and $\pi$* antibonding orbitals separated by an energy gap of about 4 eV^37^. This could lead to extended states along the helical axis with a reduced DNA energy gap. The strength of the coupling, in turn, depends on the twist angle and the separation of two successive base pairs^165^.

The appearance of a super diamagnetism is due to the ability of the material to create supercurrents^166^. These currents of electron pairs do not dissipate energy and can persist forever without obeying the Joule Effect of energy loss due to heat generation^91,167^. The currents create the strong magnetic field necessary to support the well-known Meissner Effect^168^. Using the spin-Hamiltonian formalism of VB Theory, Kuwajima proposed that a collective circular flow of the electrons across the perimeter of the aromatic ring in the spin-alternant state could exert diamagnetic susceptibilities^47^. It suggested a link between the delocalization mode and aromatic compounds' magnetic properties. An enhanced diamagnetism of aromatic compounds perpendicular to the plane of the ring has been described^47,72,169^.

Solid-state systems qubits employed in superconducting devices lose coherence on a very short time scale due to the strong interaction with the environment. The coherence time should be 10^4^ times longer than necessary to finish one operation. The coherence time in a Josephson Junction is closely related to its quality^170^ The Josephson Junction described in this work is formed by an H-bond between two nitrogenous bases (NH–N). Tunneling of interstitial H between adjacent O sites (O–H–O configuration) has been observed with a typical O–H bond length of ~ 1 Å^171^. At specific O–O distances, tunneling between two symmetric two-level systems positions have been demonstrated with high quality^172^.

When the two superconductors are separated by an insulating medium of a few nanometers, the Cooper pairs can cross the barrier by the Tunnel effect and maintain their phase coherence^110^. Some superconductor/superfluid systems with a separating constriction/insulator, generally known as “weak links” with similar current-phase relationships, show the ideal Josephson Effect^173,174^. Van der Waals-Josephson Junctions can accelerate the development of advanced superconducting devices, and Josephson-Junction qubits have potential applications as building blocks for quantum computing^175^. The dimensions of the weak link need to be comparable to the minimal length over which the wave function can change^108,174,176^. Two-qubit gates have been implemented with Josephson-Junction qubits^177^. When judging how suitable a physical system is for building a universal digital quantum computer, the gold standard is the DiVincenzo Criteria^178,179^. DNA fulfills these criteria since it is possible to fabricate multiple qubits, initialize them to a simple, known state, and perform both single- and two-qubit gates on the qubits with high fidelity without losing the quantum coherence. In addition, it is possible to measure the states of the qubits.

Even under high temperatures (90 °C), DNA is the most stable molecule^180^. There has been significant interest in developing inorganic vis-à-vis bio-organic conjugates using the unique properties of DNA. The results are extremely noisy, and simulations always depend on arbitrary conditions. For example, using a reduced model size or single-strand DNA^180,181^, DNA types^182–184^, and DNA analysis in non-physiological environments^183^. DNA is a fascinating molecule and one of the most complex systems. Although theoretical methods can help gain a detailed understanding of DNA structural and dynamic properties, their practical use is problematic due to DNA’s multiscale nature, which is characterized by the interactions of many components. In our work, we have only described the primary structure of the molecule at some point. The three-dimensional structure with the spatial configuration and the association with protein complexes make reproducing the physiological and structural model a challenge. A fundamental problem could be the degrees of freedom of the bases from their equilibrium positions. Also, to simulate natural conditions, it will be necessary to incorporate other parameters like the viscosity and nucleus environment into the model.

Some designs were described to improve the base-pair interaction modeling. Although the Peyrard–Bishop model, a well-known example of the translational model, has demonstrated an advantage over those assuming harmonic approximation^185^, the temperature dependencies of some parameters are not completely clear. In general, classical molecular dynamics simulations can only access microsecond timescales. Fully atomistic descriptions reproduce the dynamics of every single atom in a DNA molecule^186^, but they do not yet account for quantum effects. Coarse-grained DNA models allow us to reach realistic DNA sizes and time scales but don’t address the proper structure. The oxDNA and more recent computational models don’t estimate DNA folding or melting^187^. The problem with mathematical approaches like the worm-like chain (WLC) is that some intrinsic parameters have not yet been experimentally determined, and we use their estimations^188^. Other techniques used to study nucleic acids include a model parameterized to reproduce melting temperatures, an abstract model for R-loop formation, and Quantum-chemical calculation. None of these tools have managed to simulate DNA's structural and functional model in natural conditions. Therefore, we believe that a better understanding of the structure by combining knowledge of chemistry, biology, and quantum physics opens new doors for developing more effective methods for studying DNA and would bring us closer to creating the perfect quantum computer.

## Conclusion

DNA is a meeting point for biology, physics, and chemistry. A quantum physical–chemical-biological knowledge of DNA as the molecule in charge of transmitting genetic information shows the complex phenomena of Theoretical Quantum Physics in forming correlated pairs. Quantum Physics recognizes two types of particle systems: fermions and bosons, both exclusive. Generally, most physical processes are obtained from systems of fermions or bosons, but mixed systems present many difficulties. In these hybrid systems, the quantum nature of bosons or fermions is not changed, but both interact to create a complex phenomenon, as in the BEC and superconductivity. In this work, we introduce the formation of correlated pairs that use a Hamiltonian with two interactions: the Coulomb repulsion and the attractive force electron-vibrational energy-electron, forming resonant oscillatory quantum states between electrons and hole pairs. BCS, London, Ginzburg–Landau, and Abrikósov's Theories are insufficient to explain superconductivity based on these pairs in DNA. A static current is obtained due to the oscillatory movement between the electron and hole pairs in a single band. The physical–mathematical model and the graphic mechanism are represented.

The formation of electron and hole pairs in molecular orbitals can occur differently depending on the internal mechanism. For example, pair formation in Benzene differs from the nitrogenous bases because N can contribute to the $\pi$-cloud with a lone pair of electrons localized in one ${p}_{z}$ orbital. Here, we present the necessary conditions for forming pairs in these aromatic compounds: 1-There must be electrons and holes in the interacting orbitals, 2-The energy difference of the electrons must be equal to the molecular lattice vibrations. The electrons have the exact momentum, P, and are in the opposite direction, only differentiating in the momentum of the Boson. The pairs behave like bosons and condensate in a $\pi$ orbital. In this paper, we conduct a theory-based study of the nitrogenous base condensation within the framework of BEC Theory. In the two-dimensional Bose systems with no internal degree of freedom, circulations of the pairs produce a supercurrent. In both Benzene and nitrogenous bases, the supercurrent of electron and hole pairs flows, forming an internal circular ring. This supercurrent is also responsible for the unique properties of these molecules in terms of stability, balance, and low chemical reactivity.

Phase transitions in two-dimensional systems with continuous symmetry have attracted much attention. This work also presents how the purine and pyrimidine bases are connected through two or three H-bonds, but only the central H-bond functions as a Josephson Junction. The effective attraction between electrons resulted from the positive pair binding energy of RAHB and may lead to various charge density-wave and magnetic states. Finally, we describe how DNA behaves like a quantum computer, defining the quantum states that form the qubit. Josephson-Junction qubits are one of the most promising platforms for quantum computation. A physical–chemical-biological knowledge of DNA will allow us to control its properties with external parameters in the future. New doors are opening within the world of nanoelectronics and nanomedicine. God created the most perfect quantum computer: the DNA.

## Data Availability

The datasets used and/or analyzed during the current study are available from the corresponding author on reasonable request.
